# Interventional Response of Hospital and Health Services to the Mental Health Effects of Viral Outbreaks on Health Professionals

**DOI:** 10.3389/fpsyt.2022.812365

**Published:** 2022-02-22

**Authors:** Grace Branjerdporn, Candice Bowman, Sean Kenworthy, Nicolas J. C. Stapelberg

**Affiliations:** ^1^Gold Coast University Hospital, Southport, QLD, Australia; ^2^Faculty of Health Science and Medicine, Bond University, Gold Coast, QLD, Australia

**Keywords:** COVID-19, mental health interventions, health service, system, pandemic, healthcare workers, psychiatric services, staff wellbeing

## Abstract

The aim of this integrative review was to examine the impact of past viral epidemics on staff mental health interventional responses, with a specific focus on healthcare provider response in the context of the COVID-19 pandemic. Following PRISMA methodology, databases were searched for relevant articles. A total of 55 articles with a range of methodologies (e.g., commentary papers, cohort studies, qualitative studies) were included to ensure broad coverage of this rapidly emerging research area. The literature showed that many healthcare providers implemented a variety of wellbeing initiatives to support their staff during a viral outbreak. Most of these interventions, however, were not formally evaluated. Interventions included leadership/team support; online psychoeducational resources and updated information on the pandemic; respite spaces; peer support outreach; staff resilience training; telephone hotline support; staff support groups; and individual counseling. Staff were generally supportive of the initiatives offered by hospital and health services, with certain interventions being more appreciated (e.g., staff respite areas). Rapid, locally, and culturally appropriate workplace-based responses may counter the negative mental health impact on staff; but a stepped response is required for a smaller number of staff at risk of mental illness, or those with pre-existing mental illness.

**Systematic Review Registration:** Unique Identifier: CRD42020222761.

## Introduction

In March 2020, the World Health Organization (WHO) declared the COVID-19 outbreak as a pandemic and warned about the impact of COVID-19 on the wellbeing of healthcare workers (1, 2). Viral outbreaks represent a significant burden to healthcare systems and healthcare workers worldwide (3–6). There is increasing evidence that viral outbreaks such as COVID-19 put enormous pressure on healthcare workers resulting in an increased prevalence of mental health issues, such as anxiety, depression, acute/posttraumatic stress, and burnout (6–11). Studies have also shown that the psychological burden of viral outbreaks disproportionally affects healthcare workers compared to the general population (12, 13). During the COVID-19 outbreak, Zhang et al. reported a significantly higher prevalence of anxiety, depression, insomnia, and obsessive-compulsive symptoms in healthcare workers compared to non-health care workers, with a subsequent study reporting similar results (12, 13).

There are many reported reasons why healthcare workers may be more susceptible to experiencing stress and mental health symptoms in response to a viral outbreak (14–18). Fear of infection and fear of infecting patients and loved ones is a major source of anxiety and may be compounded by shortages of personal protective equipment (PPE) (15, 16, 19). Apart from a fear of illness or death due to infection, the socioeconomic uncertainty related to self-isolation may also contribute to the fear of infection (20). A substantial stressor within the workplace is the moral injury that may be caused by having to allocate insufficient resources to patients in equal need (14) or sending people home to die [e.g., (21)]. Healthcare workers may even face increased stress outside the workforce due to the stigmatization, avoidance behaviors, and apathy toward public health measures by the public (18, 22).

The National Institute for Health and Care Excellence (NICE) recommends “active monitoring” of healthcare workers as an important secondary prevention measure for mental health issues, such as post-traumatic stress disorder (PTSD) (23). The Australian Government COVID-19 mental health plan (24) also recommends developing strategies to support the needs of the healthcare workforce. While the primary and secondary prevention of mental health issues are integral to reducing their incidence, there is a clear need for appropriate interventions when these symptoms do arise. External and dedicated psychological support for healthcare workers may be critically important to improving mental health outcomes especially in a crisis response situation such as a viral outbreak (1).

The aim of this integrative review was to (1) explore the interventional responses that healthcare providers have provided to support the wellbeing of health professionals during viral outbreaks (the COVID-19 pandemic as well as epidemics such as SARS); and (2) examine how the crisis interventional responses were implemented by hospital and health services (e.g., adapt existing support services or establish new support services), including barriers to implementation and participant access. This work is important for informing recommendations to support healthcare workers during and after the COVID-19 pandemic and has overall implications for the ongoing maintenance of the healthcare workforce and patient care (1).

## Methods

### Aims

The aim of this integrative review was to (1) explore the interventional responses that hospital and health services have initiated to support the mental health of health professionals during viral outbreaks (e.g., mental health first aid, telephone hotlines, etc.); and (2) examine how the crisis interventional responses were implemented by hospital and health services (e.g., adapt existing support services or establish new support services). The review was registered on November 27th 2020 on the International Prospective Register of Systematic Reviews (PROSPERO) prior to commencement: CRD42020222761 (34TU https://www.crd.york.ac.uk/prospero/display_record.php?ID=CRD42020222761U34T).

### Design

This integrative review was conducted using a methodology framework proposed by Whittemore and Knafl (25) comprising five stages: problem identification, literature search, data evaluation, data analysis, and presentation. The framework allows for inclusion of a combination of research methodologies including quantitative and qualitative study designs such cohort studies, case-control and cross-sectional research (25). This integrated review methodology was used to provide a more comprehensive understanding of the interventional responses that have been completed by hospital and health services to support the mental health of health professionals during viral outbreaks. We used a single matrix to synthesis information, allowing for the iterative process of detecting emerging patterns and commonalities of the included articles (25). Themes are derived based on similar data being extrapolated, reduced, and categorized for analysis. These themes inform the overall summary as conclusions are drawn from each theme (25).

### Search Strategy

A systematic search of the literature was conducted with six electronic databases being searched in February 2021 including PubMed/MEDLINE, Embase, CINAHL, PsycINFO, and Web of Science. The hospital librarian assisted in selection of the search terms and development of the search query. The search query was developed by combining several criteria including (1) viral outbreaks (e.g., Influenza, coronavirus, MERS, SARS), (2) mental health (e.g., mental disorder, depression, anxiety, PTSD, stress) (3) intervention (e.g., mental health services, counseling, crisis response) and (4) healthcare staff (e.g., healthcare workers, professionals, practitioners). Search terms were combined with the Boolean operator “AND” as the search connector and “OR” in the combinations of the descriptors. An example search strategy can be seen in Supplementary Table 1. Search results were filtered to only include papers published from 2000, in English and with human subjects. The reference lists of all included studies were manually searched for additional relevant publications or key terms (26).

### Inclusion and Exclusion Criteria

All empirical evidence of implemented interventions both quantitative and qualitative (e.g., cohort studies, case-control, cross-sectional research) were included in the review. Gray literature and non-empirical publications such as theoretical papers, and papers providing only recommendations or proposed interventions were excluded. Papers were eligible for inclusion if psychological interventions for healthcare workers in response to a viral outbreak were examined (and outcomes of the intervention were discussed). Interventions not directly addressing mental health such as increasing PPE or clearer protocols were excluded. Interventional responses had to be initiated and delivered by a healthcare provider to healthcare workers (employed at a hospital and who work onsite at a hospital or in the community) during a viral outbreak. Papers pertaining to Ebola or HIV outbreaks were excluded due to low transmission rates.

### Search Procedure and Outcomes

All database search results were imported into Covidence systematic review software (Veritas Health Innovation, Melbourne, Australia, available at 34T www.covidence.org 34T), via Endnote (Version 9). The Covidence software was used to manage papers during abstract/title screening, full text review, and data extraction. Duplicates were removed in either Endnote or Covidence. Once duplicates were removed, each title/abstract was screened based on the eligibility criteria by two student researchers independently, with a separate researcher to adjudicate differences. From there, two researchers (GB, CB) independently screened the full text, with a third senior researcher mediating for any discrepancies. All authors agreed on papers included in the full review.

### Strength of the Evidence

To date, there is no gold standard for assessing methodological quality in an integrative review (25, 26). Therefore, several critical appraisal tools were used based on the methodological design of the article. The National Health, Lung, and Blood Institute (NHLBI) (27) quality assessment tool for observational cohort and cross-sectional studies, as well as the tool for pre-post studies and case-control studies was used. The NHLBI tools assess a range of parameters, such as clear and defined study population and the frequency of controlling for confounding variables. The Critical Appraisal Skills Programme (CASP) qualitative checklist (28) was used for qualitative studies. This 10-item checklist evaluates the level of bias and quality of qualitative studies. The Joanna Briggs Institute (JBI) critical appraisal checklist for text and opinion papers (29) was used for commentary/editorial papers. The 6-item JBI checklist examines the source of the commentary, whether the article discusses the relevant population of interest, the logical analytical process of the commentary, and whether it refers to the extant literature. A rating system (“yes,” “no,” “not applicable,” “undecided”) was used for each criterion. Two authors independently assessed the quality of the eligible articles (CB and GB). Any potential disagreements were adjudicated by a third author (CS) where necessary. All papers were included in the review regardless of methodological quality (25).

### Data Extraction and Synthesis

The Whittemore and Knafl (25) method of data extraction was employed. Similar data were grouped into systematic categories, and themes and relationships were analyzed. Key aspects of the included studies (supporting the narrative of the data) are shown in Supplementary Table 1 (25). The main characteristics of these studies were rigorously extracted by the authors, and any discrepancies were resolved by discussion between CB and GB. In the event of a disagreement, a third reviewer (CS) was consulted (25).

## Results

### Search Results

An adapted Prisma Flow Chart (30) is presented in Supplementary Figure 1. Our search results produced 7,944 potentially relevant articles. Six articles were also identified from a manual hand search of the literature. Titles and abstracts of the articles were reviewed: 2,450 were duplicates, and 4,954 were excluded after not meeting the inclusion criteria. The full text of the remaining articles was reviewed, and 491 articles were excluded for the following reasons: two articles were duplicates, seven were not in English, 20 examined the wrong population, and 462 did not describe an intervention conducted. Finally, 55 articles were selected for inclusion. For each study, information was collected about the author(s), year of publication, viral epidemic, article type, population identified, and main themes/results. The data of the included studies were extracted and summarized (see Supplementary Table 1).

### Quality Assessment

Quality assessment of the included studies are shown in Supplementary Tables 2–6. For the NHLBI (cohort and cross-sectional studies), all 14 studies had clearly stated the study objective, and defined the study population. The studies, however, were heterogenous in nature. Most studies had a participation rate of at least 50% and participants were recruited from the same or similar populations. Most studies did not examine the exposure of interest prior to the outcome. The studies did not control for potential confounding variables. All used valid measures and had low attrition rates but did not blind participants.

For the CASP qualitative checklist, all six studies had a clear statement of aims, were appropriate for qualitative methodology, had an appropriate recruitment strategy, and included sufficient data. These studies, however, were also heterogenous in nature. The studies did not consider the relationship between the participants and the researcher, and most did not consider ethical issues. One article was a case-control study (assessed by NHLBI case-control appraisal tool) and three articles were pre-post studies (assessed by NHLBI pre-post studies tool). These studies clearly stated the objective of the study, clearly described the intervention delivered, but the assessors were not blinded to the outcomes and the sample sizes were quite small.

Most of the included articles were text and opinion papers (31 of 55 articles). For the JBI checklist for text and opinion papers, all articles clearly identified the authors, and the authors had a standing in their field of expertise. All articles addressed the interests (i.e., mental health impact) of the relevant populations (e.g., vulnerable populations). In addition, most articles referred to the extant literature and some noted limitations with their positions.

### Identified Themes

Five key themes were identified in the included literature, comprising staff support at work, staff resilience training, staff support groups, telephone support, and individual mental health support. The extracted thematic categories are presented in Table 1.

**Table 1:** Most common themes in the literature.

**Staff wellness interventions**	**Description**	**Articles**	**Summary**
Interventions to support staff at work	Daily communication Leadership and team support Online psychoeducation (e.g., websites, mobile apps) Safe spaces Free meals, accommodation, childcare, parking Virtual social activities for staff Ward rounding by mental health professionals Staff encouragement and community support Clergy support Mourning rooms	28 articles: 21 commentary articles 5 cross sectional studies 2 qualitative studies	Staff respite spaces were highly utilized and appreciated by staff.
Staff resilience training	Resilience training Exercise intervention Coping strategies Debriefing groups	12 articles: 7 commentary articles 2 qualitative studies 1 pilot study 1 cross-sectional study 1 case-control study	Reliance training helped staff cope with the outbreak and positive feedback was reported
Staff support groups	Staff support groups Virtual drop-in centers Peer support and outreach	23 articles: 14 commentary papers 5 cross-sectional studies 3 qualitative studies 1 pilot study	In these support groups, staff expressed their fears, anger, and fatigue about the outbreak. The groups were positively received by staff.
Telephone support	General support via telephone hotline Crisis hotline Mental health screening and follow-up support Referral to mental health services	13 articles: 9 commentary articles 2 ecological studies 1 qualitative study 1 cross-sectional study	Most calls were related to requests for information about the outbreak, fear and concern about the contagion, and requests for further psychological support.
Individual mental health support	Psychological first aid One-on-one peer support Individual psychotherapy (e.g., CBT, ACT, music therapy) Consultation psychiatry Onsite crisis intervention	22 articles: 15 commentary articles 1 pilot study 6 cross-sectional studies	A stepped-care approach was implemented starting with initial screening of psychological problems. One-on-one support offered to those who needed it. Nurses were more likely to need psychological support.

### Staff Interventions

#### Interventions to Support Staff at Work

Twenty-eight papers describe how hospital and health services provided general support to staff at work. Twenty-one papers were commentary articles (including a newsletter), five papers were cross-sectional studies, and two papers were qualitative studies. General support services included daily communication (31–33); leadership and team support (31–40); a dedicated website with information about the outbreak and resources on mental health (36, 37, 41–45); mobile phone applications (37, 46–49), staff respite spaces (32, 34, 41, 42, 44, 49–54); free food, accommodation, parking or childcare; staff motivational messages (33–35, 39, 41, 43, 44, 49, 55, 56); virtual social activities for staff (31, 34, 56); on-the-ground ward rounding (36, 44, 52, 55, 57); and clergy support (41). In Australia, Poonian et al. (32) also reported how staff were rotated between higher and lower demand areas to prevent fatigue and burnout; staff were not rostered for more than four consecutive shift. Similarly in New York City, the Helping Healers program (a support group for healthcare workers) was implemented to make wellness rounds and to offer peer support to frontline staff (57). In Wuhan China, a hospital service treating severe patients with COVID-19 implemented eight measures to help reduce stress in ICU nurses, including relaxation exercises under the guidance of mental health professionals, peer support, psychological assessment, and referral to mental health support if needed (39).

In summary, support initiatives offered to staff at work were appreciated and generally utilized, but there were some barriers to participation. Gutkin et al. (56) reported that 36 (67%) of individuals agreed that the interventions provided helped boosted staff morale during the pandemic; 30 (56%) responded that the interventions were “just right” (in comparison to “not enough” or “too much”), with the gift cards being the most appreciated; and 32 (59%) found the weekly podcasts were very helpful (56). In contrast, the Zoom drop-in centers was rated as the least helpful intervention, likely due to the lack of facilitation in the groups (56). Bernstein et al. (41) reported that the most utilized intervention was the staff support centers, while the least utilized intervention was the clergy support. The authors reported that to date, many of the interventions are still ongoing, except for the staff emotional phone line due to lack of use (41). Barriers to participation include lack of time, lack of awareness of services available, and lack of support from upper management (41). The authors also noted that non-clinical staff (who were more likely to be people of color and have lower incomes) were not offered these interventions (41). Krystal et al. (35) did not report any evaluation data but did describe lessons learnt from the tiered interventions. These lessons included the dwindling of attendance at the general town halls (but not the dedicated town halls) and the stigma associated with staff seeking help for mental health programs (including fear of being reported to Department of Health) (35). Davis et al. (58) reported that no one (i.e., nurses in psycho-oncology services) utilized the one-on-one specialist sessions offered by clinician psychologists. The authors suggested that this may be due to the reduction of services at the hospital. Likewise, Gonzalez et al. (55) reported that staff were less likely to participate in the virtual support groups offered but they were more likely to participate if the groups were identified as “Daily Mindfulness and Mediation” groups.

In China, a multifactorial psychological program was implemented and then modified to accommodate the needs of the health care workers during the COVID-19 pandemic (54). Based on feedback received, it was noted that staff were reluctant to participate in the interventions as staff denied psychological difficulties; they were more concerned about their families being infected by them, about PPE shortages, and about feelings of incapability when caring for critically ill patients or those who were unwilling to comply with medical directives (54). Staff, instead, voiced that they preferred more uninterrupted rest, PPE, mental health support for patients, and skills to support patient's psychological difficulties (54). Consequently, in the second phase, the hospital modified the psychological intervention so that there was a safe place for staff to isolate away from their family so to prevent passing on the infection to them. This paper illustrates that having an assertive support approach (from telephone to face-to-face support in a rest area), which addresses the practical needs of health care workers, is what is required (54). Limitations of this paper include baseline and post-intervention measures not being implemented to assess the change in distress due to the intervention, and final evaluation of the second phase not being reported (54).

Likewise in Wuhan China, Cheng et al. (31) described a daily questionnaire that was used to monitor the daily mood of staff (the overall mood index was then broadcasted to all staff in order to provide positive self-affirmation and to promote resilience). Cheng et al. (31) reported that staff maintained an overall positive attitude (7–9 out of 10) across a 6-week period during the early stages of the pandemic (February–April 2020). Out of the 155 medical staff, 48 individuals completed the daily mood report, 65 individuals attended the daily chat group, 123 listened to the daily mood broadcasts, and 65 individuals participated in the weekly groups (31). Cheng et al. also reported the follow-up evaluation where all 155 staff members were sent an online questionnaire rating the interventions. A total of 124 staff members completed the follow-up questionnaire (41 doctors, 79 nurses, and 4 administrative staff) (31). The findings showed that 99.2% of the participants followed the daily mood broadcast, 52.4% were involved in the daily chats, and 27.4% participated in the weekly group chats (31). The findings also showed positive evaluations of the weekly groups and the daily chat groups (31). Moreover, the number of severe COVID-19 cases had a negative impact on the variation of daily mood (31). Cheng et al. (31) noted the lack of control group and the use of non-standardized measures as limitations of the study.

During the SARS outbreak in Toronto Canada, Maunder et al. (52) visited the Mount Sinai hospital (Toronto, Canada) to interview patients and staff on their experiences of SARS. In the 4-week study period, 8 patients and 11 staff members were infected with SARS. Overall, staff reported being overly concerned with being infected with SARS and/or passing SARS to family, friends, or colleagues. In particular, Maunder et al. described the hospital environment as one being a place of uncertainty and stigmatization (for patients and staff). The hospital provided psychological support to both patients and staff (52). On the SARS units, flyers were distributed to front-line staff that provided information on how to identify signs of anxiety and stress, and where they can seek help and further support resources (52). Psychiatric staff also offered informal support to staff on the ground while visiting SARS patients (52). The hospital also established a drop-in support center for staff, where telephone hotline was made available for staff who needed confidential psychological support (hotline was managed by inpatient psychiatric nurses) (52). The setting of the drop-in space was set up to be a calming space, with soothing music, comfortable chairs, and refreshments available (52). No formal evaluation (including utilization rates) was reported in this paper.

Several articles describe dedicated staff respite spaces. In the US, Ey et al. (50) describe their resident and faculty wellness program—a program initially designed to support the wellbeing of physicians (established 16 years ago) but was now used to support staff during the COVID-19 pandemic. New initiatives for mental health support for healthcare workers include staff respite spaces (including yoga mats, refreshments) and a resilience training consultation for team leaders (50). The authors reported a downturn in the utilization of the resident and faculty wellness program during the early stages of the COVID-19 pandemic, but utilization returned to normal and above baseline levels seen in previous years (18% over the past year) (50). Original support services provided by the resident and faculty wellness program were also switched to virtual telehealth at the beginning of the pandemic (50).

In the UK, Blake et al. (51) described two wellbeing centers implemented at an acute hospital trust. The authors examined the attendance of the wellbeing centers across a 17-week period (6P^th^P April to 31P^st^P July 2020). A total of 819 of hospital staff members completed the survey (55.2% of these participants had accessed the wellbeing center) (51). The findings revealed that there was moderate-to-high job stressfulness, low wellbeing, and high presenteeism (51). Moreover, a third of the staff members reported having intentions to leave the hospital (51). The findings also revealed that wellbeing was higher in those who visited the wellbeing centers than those who did not (51). During the initial stages of the pandemic, the wellness centers were highly utilized by staff (51). But as the COVID-19 admissions subsided, the utilization of the wellness centers declined, but remained at a steady flow (51). The wellbeing centers were mostly accessed by frontline health care workers and least accessed by non-clinical staff (51). Barriers in accessing the centers for non-clinical staff members included being unaware that the wellness centers were available to them, and the belief that they did not deserve the space as much as the clinical staff members (51). Ambulance workers and doctors in training were also not likely to access the wellbeing centers due to being more likely to be working offsite (51). The most common reported reasons for using the wellbeing centers included being able to rest and to have a break; for social contact and peer support; and to access a wellbeing buddy (51). The authors noted the significant financial and human resources in running the wellbeing centers and questioned the long-term sustainability of these centers (51). The authors also noted some limitations of the study including repeated visits not being recorded; monitoring data (e.g., primary reason for attendance, ethnicity details) being captured by the wellbeing buddies, which may limited the accuracy of the data collection and bias in responses; and challenges in filling in the forms by the wellbeing buddies due to supporting the person at that moment (51).

Some articles also discussed online platforms and mobile phone apps to support staff during the COVID-19 pandemic. Liu et al. (59) reported that medical staff in China received mental health education via a number of online platforms, including WeChat, Weibo and TikTok. Messages posted on Chinese blogging websites were also screened by artificial intelligence software to identify those experiencing suicidal ideation and to notify volunteer mental health workers of these situations (59). In the US, DePierro et al. (49) developed a mental health app for staff to self-screen for mental health symptoms, to track their progress overtime, and allow for the staff member to access the hospital's wellness resources (49). The University of North Carolina School of Medicine also implemented a mobile application called Heroes Health, which aimed to support health care workers' mental health (47). The free app assesses health care workers' mental health symptoms through weekly questionnaires, provides reports on mental health symptoms over time, alerts organizational leaders when additional support is required, and provides mental health resources (47). While this app has been well-received by staff, there is limited data about the uptake of this intervention (47).

#### Staff Resilience Training

A total of 12 papers discussed resilience training for staff. Seven papers were commentary articles, two papers were qualitative studies, one paper was a pilot intervention study (pre-post), one paper was a cross-sectional study, and one was a case-control study. Resilience training included strategies to improve coping skills (49, 50, 54, 60–65), stress awareness (60, 63), relaxation (54, 60, 63, 64, 66), mindfulness (60, 61), and self-care (60, 65, 66). Most of the training programs were described in detail but not evaluated for effectiveness.

For instance, Hall et al. (60) describe the Stress Management and Resiliency Training—Relaxation Response Resiliency Program (SMART-3RP) that was implemented in a hospital in Boston (MA, USA). The resiliency program consisted of an intake session and eight 90-min sessions, delivered by a trained facilitator to small groups on weekly basis (60). Both didactic and skills training was incorporated to improve stress awareness, improve adaptive coping skills, and improve the relaxation response (e.g., mindfulness, deep breathing, self-empathy, humor, creativity) (60). This multimodal, mind-body approach emphases the dynamic interplay between physiological factors, cognitive-behavioral factors, social support, health behaviors, and stress buffering skills in the midst of COVID-19 (60). Anecdotal feedback from participants showed that staff appreciated the peer support (60). Further research is required to examine mental health changes with psychometric assessments and the longer-term benefits of the training (60).

Three papers formally evaluated the utilization and effectiveness of the staff resilience programs. Aiello et al. (62) described a 1-h training session provided to health professionals, which aimed to support resilience in staff for future viral outbreaks. A total of 1,250 staff attended the training across 22 departments and were asked to complete both verbal and written feedback. Of the 1,020 that returned the evaluations, 76.1% perceived that they were better able to cope in a pandemic, and 91.8% perceived the session was relevant to work life (62). Some results from qualitative data suggested the need for timely and reliable information, strong organizational leadership, and further resilience training (62). Increased research is required to explore longer-term outcomes of the training (62). Likewise, In Haifa Israel, Amiel and Ulitzur (63) implemented a basic resilience program of 3 weekly sessions in the context of COVID-19. The workshops were designed to support surgeons and residents to understand compassion, fatigue, and the impact of stress, as well as learn spiritual care tools as coping strategies such as peer support, conscious breathing, and deep listening (63). Most surgeons (*n* = 20, 94%) who participated reported that the sessions were valued and that they desired further sessions (63). Residents (*n* = 8) who attended also reported positive effects 3 months following the workshops (63). Further research is required to examine the translation of these skills during the pandemic (63).

In Spain, Rodriguez et al. (66) described an on-site brief mindfulness program that was conducted with healthcare workers of in a public hospital in Madrid during COVID-19. The program consisted of 5–10 min of mindfulness practices delivered twice daily by psychiatrists, psychologists and mental health nurses who were supervised by certified mindfulness trainers (66). It was explained to staff that self-care was important, that it was as necessary as PPE, and that this inner calmness may be drawn from in times of the storm (66). Subsequently, there was emphasis on focused attention on an anchor, conscious movement such as soft hatta yoga stretching exercises, and compassion (66). The intervention was flexible to the needs of the group, and generated an internal pause (66). Approximately 46% of participants were nurses and staff came from: Intensive Care Units (23%), COVID-19 Medical Units (38%), and the Emergency Department (22%) (66). While 1,000 staff participated in at least one session, 150 participants reported that the sessions were 8.4 out of 10 for stress reduction across a 3-day data collection period (66).

Staff resilience training has also been incorporated as a part of staff debriefing sessions. Azizoddin et al. (67) outlined an interprofessional debriefing program for two emergency departments (ED) in Boston (USA). The Brigham Resilience in COVID-19-pandemic Emergency Forum-BRIEF was an opportunity for ED staff to debrief in nightly, video-conferencing sessions (30-min each) across 6 weeks (67). Overall, 81 staff participated across the sessions (67). A total of 24 (47%) of the available sessions were attended, which increased in higher COVID-9 presentations (67). An average of three participants attended each session (range = 2–8) and all professions were represented (67). Topics discussed included challenges of social distancing, scarce resources, and clinician burnout (67). Lessons learnt from debriefing were provided to executive management, which supported quality improvement and greater mental health support (67). Further research is required to assess the change in mental health outcomes due to intervention, and compare with those not receiving the intervention (67).

Staff resilience training has been delivered using online platforms. In Ontario, Canada, Maunder et al. (64) examined the benefits of a computer-assisted resilience training to prepare healthcare workers for the influenza pandemic in a randomized trial that compared dosage. There were three dosages of the training that had varying median cumulative duration: short (7 sessions, 111 min), medium (12 sessions, 158 min), or long (17 sessions, 223 min) (64). Relaxation skills of relaxation, breathing, and imagery were taught using audio modules. There were also interactive reflective exercises that contained simulation scenarios and facilitated participants to consider their response (64). A range of psychometric measures were used to evaluate the effectiveness (viz., Pandemic Self-Efficacy, Inventory of Interpersonal Problems, Ways of Coping Inventory). A total of 158 multidisciplinary staff were randomly assigned to training (short, medium, or long sessions) (64). Results demonstrated that confidence in support and training, that pandemic self-efficacy and interpersonal problems improved, and that there was higher use of adaptive coping strategies (64). Medium dosage appeared to be best suited based on the resiliency outcomes and drop-out rate (64). Further research is required to understand the longer-term benefits of such training and the use of the skills in a pandemic (64).

Likewise In New York (US), DeCaporale-Ryan et al. (65) describe a videoconferencing-based training program. The sessions consisted of 20 min of psychoeducation about the likelihood and management of delirium, and 40 min of discussion to brainstorm interventions to reduce loneliness, acknowledge challenges (e.g., anxiety for patients, concern for safety of self and loved ones), offer support to each other, suggest coping strategies and to validate feelings (65). Staff from skilled nursing facilities and psychiatric centers affiliated with the University of Rochester Geriatric Telepsychiatry Program were invited to participate (65). In the early phase of the pandemic, two videoconference sessions were offered with 67 then 61 participants, respectively, over both sessions (65). Based on qualitative reports, participants found the session to be beneficial for self-care, affirming of their experience, and helpful for their patient care (65). Despite this, quantitative evaluation such as pre-/post-tests were not conducted using psychometric mental health assessments (65). As a follow-on from this intervention, sessions regarding isolation, depression management, and staff burn-out prevention were provided. However, evaluation of the second phase was not reported (65).

Finally, one study implemented a novel exercised-based intervention to improve staff mental state and wellbeing in China during the COVID-19 pandemic. In a case-control design, Wu and Wei (68) examined the effect of a novel exercise-based intervention on the mental state and sleep status of frontline medical staff (68). A total of 60 medical professionals and aged-matched controls were included in the study. Participants were prescribed a self-exercising rehabilitation which was completed at home (e.g., yoga, tai chi, qi gong) and of suitable intensity (68). Compared to controls, participants to the experimental group reported less somatization symptoms, terror, depression, post-traumatic stress symptoms and sleep disturbances post-intervention (68). The study provides empirical evidence of the benefits of an exercise-based programme in a rigorous methodology (i.e., case-control), offering between-group comparisons (68). Shortcomings of the study is that more details of the exercise intervention is needed, including information on frequency, fidelity, length of time, timing, personnel administering intervention, and the nature of the exercises; clarity regarding the timing of the post-intervention assessments is also needed, and longer-term follow-up is prudent (68).

#### Staff Support Groups

A total of 23 articles discussed staff support groups at the hospital. Fourteen articles were commentary papers (including one newsletter), five papers were cross-sectional studies, three papers were qualitative studies, and one paper was a pilot study of an intervention (pre and post). Support groups included team support sessions (39, 41, 54) drop-in virtual support groups (32–34, 43, 56, 69, 70), weekly groups led by a mental health professional (31, 42, 44, 45, 71), debriefing groups (38, 40, 72, 73), and peer support groups (32, 35, 39, 46, 74, 75).

Most of the papers described the support groups but did not evaluate the utilization or the effectiveness of the groups. In response to SARS in 2003, a psychiatric team provided support to staff in a Taiwanese hospital dedicated to caring for SARS patients (40). The SARS medical nurses suggested improvements for the psychiatric services intervention, including being more flexible, informal and relaxing to suit their work schedules; offering more individual counseling on personal issues; the debriefing groups to be small and shorter; and for psychiatric services to be ongoing as secondary traumatisation lingered past the outbreak. Likewise, during the SARS epidemic in Hong Kong, Chan and Huak (74) describe how their peer support program (aimed to provide psychological support to staff after an assault) played a role in supporting staff during the outbreak. In response to the COVID-19 pandemic, Datta et al. (75) describe several staff support groups that were held to help educate staff on infection control and PPE, and to mitigate fears and anxieties of the pandemic in Kolkata (India) (75). Similarly in New York, peer support groups were offered to staff to support them during the pandemic (69). The peer support (virtual) groups were held weekly for 40 min each, and discussions were around thoughts and feelings as a result of the outbreak (69). Joseph (46) also described a joint initiative—the Wellbeing Initiative, designed to support the health and resiliency of nurses in the US. The Wellbeing Initiative comprises of a virtual platform for peer support between nurse to nurse (Nurses Together Connecting Through Conversations), as well as mental health resources (46). The virtual forum is hosted through Zoom meetings where nurses can share and ask questions (46). Nurses may volunteer to be facilitators or co-facilitators (46). Lastly at a Children's Hospital in New York, Schulte et al. (38) implemented support video calls to reduce distress in staff during COVID-19. The calls were conducted in a group setting and lasted ~1 h. These calls were voluntary, optional, and facilitated by an executive coach certified by the board of the hospital (38). In a 2-week period, six video support calls were provided. On average, eight staff members attended each of the group calls and 48 (21%) of the hospital departments participated in at least one of the calls (38). Predominately females joined the calls, all levels of staff members were represented, and most called from home (38).

One paper evaluated the utilization and effectiveness of the groups. Mellins et al. (45) describe the CopeColumbia peer support program, which consist of peer support groups, individual sessions, virtual presentations, and psychoeducational resources (via a website) for healthcare workers. This program was specifically created in response to the COVID-19 pandemic, and the program is facilitated by mental health staff (including psychologists and psychiatrists) (45). The peer support groups were 30-min structured sessions delivered virtually to staff and were aimed to provide a safe space for staff to discuss their concerns about the COVID-19 pandemic (45). To address larger audiences, virtual presentations (conducted at Town Halls) around managing stress and anxiety were delivered (45). Between March and June 2020, Mellins et al. (45) reported 186 groups being held (1–30 participants), with 30 groups meeting just once, 22 groups meeting two-four times, and nine groups meeting more than five times. A post-group survey (124 participants) revealed the helpfulness of the groups, with 76% of participants rating helpfulness as “quite a bit” to “extremely” helpful (45). The authors note, however, that it cannot be determine as to whether the survey responses were from 124 unique participants (due to the anonymity of the survey). Response rate can't also be established (45).

Another paper described the common themes observed in staff support groups in a qualitative analysis. During the SARS outbreak, Khee et al. (71) implemented group therapy sessions with staff at the Tan Tock Send Hospital in Singapore. Most of the therapy groups comprised nurses and physicians, especially those working in ICU and in the frontline. Each session went for around 1–2 h, and staff members were encouraged to express their thoughts and feelings about the SARS outbreak (there was no structure to the therapy groups). A total of 16 therapy groups were conducted with 188 healthcare workers (71). Khee et al. (71) reported two main trends in these discussion groups: fear, anger, and blame being the predominant emotions expressed at the beginning of the outbreak; and a sense of grief and frustration after the death toll had risen (including the death of colleagues).

Two papers described and reported on staff debriefing groups. In Italy, Siracusano et al. (73) implemented an Emotional Defusing intervention for healthcare workers, which consists of discussion groups that focuses on reducing the impact of a potential traumatic event (e.g., COVID-19 pandemic) on wellbeing. Siracusano et al. (73) conducted 19 defusing groups between 16P^th^P March and 29P^th^P April 2020 in Italy. A total of 189 healthcare workers participated in the groups (70.9% females). A questionnaire was also distributed to participants in the groups that measured their concerns around COVID-19 outbreak (73). Most participants were preoccupied with the current pandemic (69.84%), were afraid of infection (62.43%), were worried about infecting family members (~45%), and reported a change in sleeping patterns (90%) (73). In the groups, the authors also noted that most healthcare workers felt stigmatized due to potential of being infected with COVID-19 (73).

Likewise in Rome (Italy), Monette et al. (72) piloted a video-based debriefing program to support ED staff during the COVID-19 pandemic. The facilitated debriefings were delivered virtually (via Zoom) and were conducted once a week for an hour each (72). Monette et al. (72) reported 18 completed Emotional Debriefing sessions, with 68 unique individuals participating. A survey was distributed to participants in the debriefing groups, asking them about their experience of the groups (72). A total of 76% of the participants completed the survey (52 out of 68 participants) and most reported their experience of the group as being positive and helpful (>80%) (72). But non-physician practitioners (78.8) reported being less comfortable in speaking up than the attending physicians (77.8) (72). The authors noted the potential limitation of selection bias into the groups, with those being more comfortable to share their feelings more likely to attend the groups than those who are less comfortable (72). In addition, the authors noted that the participants relationship with the facilitators may have biased (either negatively or positively) the survey responses (72).

#### Telephone Support

Thirteen papers discussed telephone hotlines to support healthcare workers during a viral outbreak. Nine of the papers are commentary articles, two papers are ecological studies, one is a qualitative study, and one is a cross-sectional study. Some health services implemented a telephone service for staff who needed general psychological support: 24/7 hotlines (50, 76, 77) or time-limited hotlines (49, 78); while others offered a telephone support line as a part of broader support services for healthcare workers (33, 37, 41, 44, 52, 54, 55, 79).

In North East England, a telephone support line was offered to healthcare workers in a publicly-funded health service consisting of ~14,000 staff members (78). Responders were primarily psychologists and counselors providing information and guidance, brief emotional support, containment and signposting to emotional support and advice, including referral to a 30 min support call with a mental health clinician if required (78). The telephone line was launched 1 day after a nationwide “lockdown” and open from 0,900 to 1,700, Monday to Friday (78). From the first 4 weeks of service implementation, 655 calls were made to the support line, with callers waiting 28 s on average and frequency of calls gradually reducing after the first week (78). Based on a content analysis of 362 call notes, 68% of calls were related to clarification of guidance (e.g., eligibility for swabbing, actions to take when family symptomatic or exposed to a COVID-19 case contact) and 29% of calls were in regard to offering support around anxiety (e.g., worried about increased risk exposure) (78). While the paper presents data about the level of access and nature of calls, further research is required regarding change in distress owing to the calls and user satisfaction reports (78).

In France, Geoffroy et al. (77) describes the process of developing a 24/7 telephone support line and highlights the numbers of calls, characteristics of callers, and reason for calling during the first 26 days of opening. The hotline provided support to 39 hospitals in a regional area, employing more than 100,000 multidisciplinary health professionals (77). While responders provided brief crisis intervention with rapid assessment of symptoms, immediate psychological support and referral to psychosocial support/therapies and psychiatric consultation, no specific crisis interventions were utilized (77). Across the study period, Geoffroy et al. (77) found that 149 calls were received and, on average, were 18.5 min long. On average, 5.73 calls were received per day, and were mainly between 0,800 and 1,400 (57%) (77). Those who called the hotline were mostly women (86%), around 32.7 years old, and were frontline healthcare workers (e.g., emergency room, registered nurses), although a large range of health professionals accessed the service (77). The primary reason for calling are related to anxiety symptoms (49%) due to the fear of being contaminated with or contaminating others with COVID-19, the social stress of feeling isolated or having intra-family tensions, and the work-related stress of changes in work and routine (77). Other reasons for calling were request for hotline information (20.8%), worries about COVID-19 (15.44%), exhaustion (11.41%), trauma reactivation (6.11%), insomnia (6.0%), anger (5.37%), depression (4.02%), and psychotic symptoms (2.01%) (77). The majority of calls (70.47%) were later referred to further psychosocial, COVID-specific, and general support such as psychology or psychiatry (29.5%), infections department (9.5%), and occupational medicine (12.4%). While this study provides rich detail into the nature of the hotline, further research exploring caller satisfaction or engagement in referrals is required (77).

In Italy, a telephone hotline was also offered surrounding psychological first aid principles (79). The telephone hotline was available to healthcare workers, as well as non-health care workers such as those in isolation, patients infected with COVID-19, close contacts, and members of the general public (79). Individuals had the option to call or email for an initial consultation with the clinical psychologist, and have a follow-up review with a psychiatrist if required (79). Across ~2 months (10 March−3 May 2020), 30 health care professionals accessed the service, of the total 135 users (79). Most users were nurses (*n* = 24), with other users being physicians (*n* = 1), social-health worker (*n* = 1), and a radiology technician (*n* = 1). A total of 46.6% (*n* = 14) of healthcare workers who accessed the service worked in a COVID-dedicated unit (79). Across all users, telephone (*n* = 126, 93.3%) was the main method of accessing the service, and psychological sessions were 29.18 (SD = 10.49) min on average (79). A total of 50.0% of health professionals called due to fear of contagion, followed by anxiety symptoms (43.3%) and loneliness (13.3%) (79). In contrast, people of the general population had less fear of contagion and more loneliness (79). Health professionals perceived that the current quality of life (QoL) was lower than both the past QoL and the imagined future QoL (79). This study provides is novel in that it supports both health care professionals and non-health care professionals, and compares the two groups (79).

In Chengdu, China, He et al. (76) described a four-tiered psychological program that targeted the general population, including health professionals. The first tier consisted of a television program airing daily to raise awareness of mental health difficulties amid COVID-19 (76). The second tier involved a 24/7 hotline service which responded to psychological questions and filtered emergent cases (76). For those requiring further support, online video calls were offered which was provided by mental health professionals as part of the third tier of support (76). In addition, on-site crisis intervention was provided to frontline medical staff based on the guidelines of Anticipate, Plan and Deter (76). The forth tier involved overall training and supervision by the leadership team to staff over the duration of the program (76). From 26 January 2020 to 26th March, there were 4,236 hotline consultations (tier two), lasting on average 11.30 min (76). The majority of callers were female (57.54%), and calls were related to emotional and behavioral disturbances (50.27%) (76). Based on the outcome of the call, 27.05% reported that their problems were solved, 56.26% experienced emotional relief, and 16.69% required further intervention (76). For tier three, there were 233 people who received online video psychotherapy (76). It should be noted, however, that figures here include not just health professionals, but the general community and patients with COVID-19 (76). For tier four, five psychological training sessions were conducted for 98 frontline medical staff (76). Strengths of the study were that the intervention combined both face-to-face and online modalities. Further research is required to explore usage of hotline and online video consultations with health care workers as a specific subpopulation (76).

#### Individual Mental Health Support

A total of 22 articles discussed individual psychological support offered to staff at the hospital. Fifteen of the articles were commentary papers, one was a pilot intervention study (pre and post), and six were cross-sectional studies. The types of individual support available include telehealth or virtual psychological sessions (33, 35, 37, 41, 43, 48, 59, 69, 76), psychological first aid (36, 43, 44, 49, 79–81), one-on-one peer support (45), one-on-one consultation with a psychologist (37, 39, 42, 79, 82, 83) or with a psychiatrist (37, 40, 79, 83, 84), brief interventions (85, 86), and crisis intervention (37, 76).

In Turkey, Dursun et al. (48) provided a multifaceted intervention with a mobile phone application to allow staff to report their concerns, to have video-conferencing sessions with psychiatrists, as well as to have access to face-to-face local support by a psychosocial team if required. The intervention was also unique in that it supported both healthcare workers and their children (48). Firstly, healthcare workers had to download a specific mobile phone app (Ruh Sagligi Destek Sistemi-Mental Health Support System) designed by the Turkish Ministry of Health onto their mobiles, which is matched with their staff ID number and provides information about their locality for local services if face-to-face support is required (48). Secondly, staff advised through live chat whom the support was for (self or child) and the nature of the problem (48). Thirdly, local psychiatrists provided videoconference consultation based on a mutually agreed upon time with the healthcare worker (or their child) needing support (48). It should be noted that there was no standardized psychological framework applied across these consults (48). Fourthly, face-to-face psychiatric evaluation and/or review by a psychosocial team was provided for crisis presentations (48). Of the 2,688 individuals who downloaded the app between April 8 and May 3 2020, a total of 879 sought a psychiatry appointment for themselves (48). Of those seeking psychiatric support, 351 healthcare workers participated in the video call with a psychiatrist, with high satisfaction reported (8.1/10 in satisfaction and 86.6% reporting that their support was responded to). As a follow-up, 48 healthcare workers (13.6%) required a face-to-face review by the same psychiatrist, and five required (1.4%) further face-to-face psychosocial support by a team (48). The predominant reason for referral was the excessive fear of contamination (*n* = 178, 50.7%), and 41.1% (*n* = 144) of staff that requested support were nurses (48). The strength of the study is that satisfaction with the service was evaluated; however, further evidence is required to understand the utility of the intervention across a longer time period (48).

In Portugal, Machado et al. (82) described a three-level mental health intervention developed to support staff during the COVID-19 pandemic. The bottom two levels (levels two and three) were implemented at the beginning of the pandemic and consisted of a telephone hotline (level three) and one-on-one psychological support (level two) (82). The services were specifically offered to staff working on the frontline (e.g., infectious diseases, intensive care, COVID-emergency ward) (82). However, Machado et al. (82) reported that no frontline staff had used the telephone hotline to request for psychological support (82). The authors also reported that no referrals were received for the level two support (one-on-one psychological sessions) (82). The authors speculated that being judged as “weak” or “fragile” by co-workers may have discouraged frontline workers in seeking psychological help (82). For level one support, the entire hospital workforce was screened for psychological distress (via a mobile survey on an App) (82). Completion of this survey was voluntary (82). Staff who are flagged (via the questionnaire) as needing further support were contacted by the psychiatric liaison team (82). For level one support, several staff members were flagged as needing psychological support and were subsequently followed up (82). Most of the staff who were flagged were not considered frontline staff (82). These staff members were suffering from psychological distress such as anxiety, insomnia, and compulsive eating (82).

In Italy, Giordano et al. (85) described a music therapy (remote) intervention aimed to improve the psychological wellbeing in frontline staff. A total of 34 staff members participated in this pilot study and received the music therapy intervention over a period of 5 weeks (at the beginning of the pandemic) (85). Staff members (14 doctors and 20 nurses) were those who were assisting COVID-19 patients; they were recruited via the dedicated hotel room offered to staff as a place to stay to help avoid passing on the infection to family members (85). Levels of tiredness, sadness, and anxiety were measured before and after the intervention (85). The music therapy consisted of playlists aimed to promote relaxation, calmness, and to reduce anxiety and stress. Participants were also interviewed by the music therapists on their listening experience (85). All playlists were customized for the participants, and listing guidelines were provided to help facilitate listening experience (e.g., “close your eyes” “focus on an image or a color”) (85). Overall, the results of the pilot study showed reduced levels of tiredness, sadness, and anxiety in staff after receiving the intervention (85). The authors highlight the utility of this cost-effective music therapy intervention for staff, which does not require much resources to implement (85). The authors also highlight the need for future studies with larger sample sizes to verify the efficacy of this intervention (85).

In a before and after intervention study in South Korea; Jo et al. (84) distributed the Korean version of Impact of Event Scale-Revised to 2,554 hospital workers at Yeungnam University Hospital (84). The questionnaire was sent via mobile phone. Hospital workers who scored higher than the cut-off score on this measure (i.e., diagnosis of PTSD) were offered further psychological screening by a psychiatrist over the telephone (i.e. mini international neuropsychiatric interview and clinical global impressions-severity scale) (84). Mental health support was then offered to staff workers, which included one-on-one psychiatric support over the phone over a period of 2 weeks (84). Initial questionnaires were repeated for all workers who received the individual sessions by the psychiatrist (84). A total of 253 workers/2,554 workers (9.9%) completed the mobile questionnaire (210 women and 43 men) (84). Higher levels of psychological distress were reported by non-medical staff and allied health staff than by doctors (84). Out of 253 workers, 54 workers (21%) obtained scores higher than the cut-off and were offered further assessments by a psychiatrist (84). A total of 15 workers/54 workers (27.8%) completed these further assessments and were offered one-on-one support by the psychiatrist (84). After the psychiatric intervention, 13 workers out of 15 workers (97%) had improved scores on impact of events scale-revised (84). However, the authors noted self-selection bias, generalisability (study was conducted at just one hospital), and response rate differences between the groups (e.g., highest response rate in women and nurses) as limitations of the study (84).

Kameno et al. (80) described a designated mental health ward dedicated to nurses who care for patients with COVID-19. All 31 nurses working in the ward completed a questionnaire assessing psychological distress (i.e., Kessler Psychological Distress Scale and four questions on sleep disturbance, alcohol use, and appetite) (80). Individual psychotherapy (i.e., psychological first aid) was offered to staff who scored higher than the cut-off score of the questionnaire. Each psychological session was 30–60 min in duration and was provided by a mental health nurse at three time points (April 2020, May 2020, and June/July 2020) (80). A total of eight staff members scored higher than the cut-off score and was offered psychological therapy (80). Three staff members out of eight (37.5%) accepted the therapy and five did not respond to the offer (80). The results showed that psychological distress in those who received the individual therapy improved across the three time points compared to those who did not received the therapy (despite being offered the support) (80). Sleep and appetite also improved across the three time points for those who received the individual therapy (80). The authors noted that this is just a pilot study and that large-scale studies (randomized) are currently needed to verify the findings (80).

In a letter to the editor, Torricelli et al. (86) describe their eye-movement desensitization and reprocessing (EMDR) intervention that they delivered to frontline staff members (around 200 healthcare workers in a period of 3.5 months) during the COVID-19 pandemic. The EMDR version that was used was designed specifically for healthcare workers to reduce psychological distress (induced by traumatic events such as COVID-19) (86). Both group and individual EMDR interventions were offered to staff by trained EMDR psychotherapists (86). Group sessions had around 12–15 participants in each and went for around 90 min (86). Individual sessions were requested by staff who needed further psychological support (86). No specific clinical outcomes were reported in this commentary, but the authors made some recommendations around the potential utility of EMDR in mitigating the impact of COVID-19 on staff mental health (86).

Finally, Buselli et al. (83) describe their PsicoCOVID19 intervention (developed by Occupational Preventative team), which is targeted toward healthcare workers during the COVID-19 pandemic in Italy. The PsicoCOVID19 has two aims: one is to monitor the healthcare workers who have suffered from previous mental health problems (prior to the pandemic), and the other is to provide psychological support to frontline healthcare workers involved in COVID-19 emergency (83). At the beginning stages of the pandemic, a dedicated email was established (managed by two psychologists) in order to allow staff to request for psychological support if they needed it (83). Callers were sent a self-assessment questionnaire on their psychological symptoms, including depression, stress, and anxiety (83). For healthcare workers with a previous history of mental health problems, psychiatric consultation was offered (83). For healthcare workers without a previous history of mental health problems, psychological consultation was provided. Information provided on the self-assessment questionnaire was sent to the consultation psychiatrist or psychologist (83). Psychiatric consultation involved assessment via Diagnostic and Statistical manual of mental disorders (83). Individuals were also categorized into two groups (COVID or non-COVID units) to determine their level of suitability or fitness to work in the frontline response (83). Pharmacological intervention as provided as needed (83). Likewise, psychological consultation categorized individuals based on their history of mental health problems prior to the pandemic and whether they were working in the COVID units or not (83). CBT was offered to healthcare workers to help staff manage their stress and anxiety related to the COVID-19 pandemic (83).

To date, 106 healthcare workers (out of total of 8,000 staff working at the hospital) requested the psychological support service (83). Forty-four of those completed the self-assessment questionnaire (83). Most healthcare workers reported mild to moderated levels of distress and anxiety, and 81% of participants reported a previous history of mental health problems (and were already being monitored by the team prior to the pandemic) (83). The authors speculated that the low completion rates on the self-assessment questionnaire may have to do with treatment staff not wanting to burden the healthcare workers with this task (and compromise therapeutic alliance as a result) (83). Staff reported being overall satisfied with PsicoCovid19 service they received at work, and appreciated being treated by people who shared a common experience with by working at a health service (83). The authors found that women nurses were more likely to request psychological support (83). The authors noted the limited sample size and generalisability of the study (83).

## Discussion

### Summary of Findings

Many hospitals and healthcare providers implemented a variety of wellbeing initiatives to support their healthcare workers during a viral outbreak. These interventions included leadership/team support; daily communiques about the pandemic; free meals, transport, childcare, and/or accommodation; online psychoeducational resources and updated information on the pandemic; respite spaces; peer support outreach; staff resilience training; telephone hotline support; staff support groups; and individual counseling. Overall, the literature supports the initial increase in anxiety and fear in healthcare staff around the viral outbreak, but this response generally does not reach the threshold for a diagnosis of a mental illness. General concerns of staff include being preoccupied with the current outbreak [e.g., (73)], being afraid of infection [e.g., (73, 78, 79)] or infecting others [e.g., (73, 77)], being stigmatized from the public due to being potentially infectious [e.g., (73)], experiencing work-related stressors around routine change [e.g., (77)], feeling socially isolated [e.g., (77, 79)], and feeling exhausted and fatigued [e.g., (77)]. Some healthcare workers were referred to one-on-one support (either online or in person) by psychiatrists or psychologists [e.g., (48, 76, 77)]. One service reported providing psychiatric consultation to staff members suffering from pre-existing mental health issues and that were known to the service (83).

Many of the interventions reported in the included articles had been implemented rapidly in response to a viral outbreak; staff support initiatives were either modified from exiting services (50, 74) or were newly created services (53). For instance, in Hong Kong, Chan and Huak (74) adapted their peer support program (aimed to support staff victims of assault) to help healthcare workers during the SARS outbreak; likewise, Ey et al. (50) modified their Resident and Faculty Wellness Program (a program initially aimed to provide accessible mental health support to physicians) to include new wellness resources (e.g., 24/7 hotline, website with updated information and resources, consultation service) to support staff during the COVID-19 pandemic. Some services sought feedback from staff prior to (and/or after) implementation of interventions. For example in China, Chen et al. (54) reported adjusting initial interventions based on feedback received from staff: staff wanted respite areas to manage their stress rather than individual counseling by a psychologist; they also wanted to be able to communicate with their families at work to alleviate family concerns; and to know how to manage patient anxiety around COVID-19 infection. Saqib and Rampal (53) asked staff in an online survey: do they feel that there is a need for the staff wellbeing hub, and if so, what was needed in the hub. Most responders supported setting up the wellbeing hub and reported a need for a calming environment for staff (53).

Most of the included articles in this review were from the US; these articles tended to describe the implementation of staff group sessions and resilience training (62, 67). Articles from China tended to describe virtual interventions such as online chats and training courses (13, 54, 59). Included articles from UK tended to describe staff respite centers and mental health first aid (42, 51). Whereas, in Italy, articles tended to describe individual interventions such as music therapy (85), and cognitive behavior therapy (83). A blend of both online and in-person, as well as individual and group-based interventions were offered, with staff appreciating the variety of options provided so they could engage based on their preferences and schedule (42, 44). Some studies escalated care from online universal support in the form of hotlines, training programs and group debriefing sessions, to face-to-face crisis intervention for more acute mental health difficulties (13, 76). Interventions offered in-person were more practical and embedded within clinical care, such as wellbeing hubs or staff rest areas (32, 52), mental health support during handovers, staff meetings or huddles (61), and practical supports (54). Online interventions had the advantage of limiting spread of infection. For more personalized and intensive support, interventions targeted at individuals enabled clinicians to divulge deeply and receive appropriate management (36, 50, 52). Group interventions were beneficial in that there was wide-spread support and engendered collegial relationships to develop between staff (38, 43).

Staff were generally supportive of the initiatives offered by hospital and health services, with respite spaces and free meals being particularly appreciated (41, 56). Some papers reported interventions not being utilized by staff, including a zoom-drop in center (56), clergy support (41), a phoneline (41), and one-one-one sessions with a clinical psychologist [e.g., (42, 82)]. Gonzalez et al. (55) reported that staff participation in virtual support groups was dependent on the name of the service (i.e., “Daily Mindfulness and Mediation” group had higher participation rates than a support group with no such name). Chen et al. (54) also reported that some nurses refused psychological support despite feeling fatigued and distressed. Services with low utilization rates were either withdrawn or modified [e.g., (41, 54)]. It should be noted that due to the rapidity with which support initiatives were implemented, key steps around service development were not reported on, or were not implemented, including planning, assessing readiness for change, evaluating the effectiveness of the intervention, and determining how to best engage staff (1). Healthcare providers may have prioritized delivery of an intervention over the formal evaluation of effectiveness and impact of it on staff (1).

Barriers to accessing interventions include a lack of support from upper management (41), not being aware of the support offered (51), stigma around requesting mental health support (35, 82), and not feeling that they deserved the support offered (51). Krystal et al. (35) also discussed the importance of privacy when offering support services to staff, as some healthcare workers may be reluctant to seek support due to fear of being reported to the Department of Health (i.e., mandatory reporting) and losing their clinical licenses as a result. Blake et al. (51) also discussed the practical issues associated with establishing respite areas in the hospital, including the lack of suitable space and the postponement of activities that usually happen in the spaces used as respite areas. Some healthcare providers reported the lack of sustainability of the services offered to staff and emphasized the need for ongoing funding to continue these support initiatives (49, 51). Future research is needed to understand the barriers to accessing support interventions for staff. The local needs of the healthcare service should also be considered.

### Recommendations

In synthesizing the emerging literature, key themes around staff interventional responses to viral epidemics/pandemics were identified. Table 2 describes these themes along with our recommendations listed beside them.

**Table 2:** Key themes around staff interventional response to virial epidemics/pandemics and our recommendations.

**Key themes around staff intervention response**	**Our recommendations**
1. Overall, there is an initial increase in anxiety and fear in staff, but this response generally does not reach the threshold for a diagnosis of a mental illness in most workers. A wide variety of staff support measures were implemented in response to the viral outbreak, but these were not always offered commensurately to staff needs and were not always structured as an escalating pathway according to mental health or wellbeing needs. 2. Effective communication in keeping staff updated with the latest information around the pandemic can help reduce anxiety in staff. There is a need to communicate to staff what support is available to them. 3. Staff respite spaces can reduce burnout in staff and promote safety and shared experiences. 4. Attitudes around mental health and stigma may vary between global cultures and may also vary within workplace cultures. 5. Some staff may be reluctant to seek mental health support due to fear of being reported on or a lack of privacy in the workplace. 6. It is important to consider the sustainability of the interventions (e.g., staff resources, hospital space, costs, evidence base of interventions). At-home based interventions such as music therapy and exercise therapy can be cost-effective initiatives for staff. 7. Staff should be provided with an opportunity to reflect on the interventions (both before and after implementation).	1. A stepped-care pathway to staff support is needed, with interventions to support staff at work to address initial concerns and anxiety, with stepped escalation to support increasing acuity and mental health needs, culminating in one-on-one mental health support (provided by psychiatrists or psychologists). 2. Ensure good and updated communication to staff about changing circumstances (e.g., an evolving pandemic). Ensure that staff are aware of all support initiatives that are available in the workplace, and additional external support (e.g. psychological). Leaders should be visible to front-line staff. 3. Ensure where possible that staff are well-rested, with access to meal breaks, respite areas, and social support as needed. Consider adapting, reforming or expanding existing staff wellbeing and care frameworks to meet current and future needs of healthcare workers 4. Reduce stigma around mental health for staff. Interventions should be tailored to local needs and be culturally appropriate. Teams should regularly discuss any concerns and anxiety staff may have related to the outbreak. 5. To remove barriers to support services, concerns about possible censure could be addressed prima-facie within health services. Psychological and psychiatric services should, where possible, be provided by services which are not linked directly to, or part of, the workplace. Confidential support should be available to staff. 6. Interventions should be sustainable long-term. Use telehealth and other technologies were appropriate to mitigate the spread of infection. 7. Demands on clinical staff will be high during a pandemic crisis, but if clinical staff have capacity to be involved in the development and implementation of support interventions, this could be beneficial. 8. Ensure training, supervision, and research opportunities are maintained throughout the outbreak.

The Australian Government COVID-19 mental health plan (24) further recommends that providers develop strategies to support the needs of staff (based on the learnings of COVID-19 pandemic response), to provide training and support to healthcare workers who rely on telehealth, to engage Indigenous health professionals and peer workers to ensure sustainability of services, and to monitor burnout and stress of frontline healthcare workers. The involvement of the Indigenous; culturally and linguistically diverse (CALD); and peer workforce in the staff interventional response to COVID-19 was a noted gap in the literature we reviewed. These groups should be considered as important stakeholders in the development and implementation of staff support initiatives (87). This would also ensure that healthcare workers who identify under these groups have access to the supports available.

### Strengths and Limitations

The current pandemic saw the rapid emergence of numerous (usually brief) publications, letters to the editor, or commentary articles on staff interventional responses during the initial stages of the COVID-19 pandemic, possibly due to the immediate desire to publish findings. We believe that it was important to capture the useful and practical advice offered in these publications, to guide service response in supporting staff. An integrative review was the most appropriate approach for this type of literature review, as it synthesizes the emerging literature on the interventions offered to healthcare workers during viral outbreaks, with a specific focus on the immediate response of healthcare services.

The main limitation of this work is that most of the included articles did not evaluate the staff interventions offered, so we are not able to comment on statistical robustness of the findings. Our included articles were also mostly non-empirical commentary papers, and the empirical articles that were included varied in design and nature (e.g., sample size, study timeframe, intervention type). In addition, our literature search may not have captured all the relevant articles available (gray literature was also excluded) due to the rapid emergence of publications on staff mental health interventions during COVID-19 pandemic. Non-English articles were not included, which could have biased the information gathered from the literature toward English speaking countries. It is also important to note that the impact of COVID-19 on staff wellbeing is complex and is likely to differ across cultures, countries, economic circumstances, and viral epidemics (e.g., SARS, COVID-19). Moreover, it is unclear whether the utlisation of staff interventions depended on how well a country or region was able to respond to outbreaks such as COVID-19 (including vaccination rollout). That said, the COVID-19 pandemic is global; therefore, some of the emerging evidence of staff interventions should be applicable to different geographical contexts (1). In addition, while this review aimed to include viral outbreaks other than COVID-19, the review was conducted within the context of the current COVID-19 pandemic. The current pandemic is still ongoing, with problematic variants emerging, making the progression of this pandemic unpredictable in the long-term. Therefore, the recommendations on supporting staff during a pandemic are to some extent limited by the timing of this review. Future research should include gray literature to better capture the health-service response to supporting staff during a viral outbreak.

## Conclusion

The integrative review showed how multiple staff support measures were implemented in response to a viral outbreak. Rapid, locally, and culturally appropriate workplace-based responses coupled with a good communication strategy may counter an initial increase in anxiety and fear in staff, but a stepped response is required for a smaller number of staff at risk of mental illness, or those with pre-existing mental illness. An escalated response to mental health assessment and care should be tailored as much as possible, avoid issues of stigma or censure, should respect confidentiality, and should be separate from the workplace and its governance. There is currently limited evidence on the effectiveness of the interventions offered to staff, and formal evaluation of the implemented initiatives were not prioritized due to the immediate response to support staff during the initial stages of the outbreak. Future rigorous research on the most effective ways to support staff during a global pandemic is urgently needed. Long-term sustainability of staff interventions (including long-term outcomes) are also needed to prepare for the long-term impacts of COVID-19 and future pandemics.

## Data Availability Statement

The original contributions presented in the study are included in the article/Supplementary Material, further inquiries can be directed to the corresponding author/s.

## Author Contributions

GB, CB, and NS conceived the idea of the article. SK, GB, and CB screened and reviewed the abstracts and articles based on the inclusion/exclusion criteria. CB and GB reviewed the selected full-text articles with NS mediating any disagreements. All authors agreed on papers included in the full review, took part in the data extraction of the common themes, involved in the drafting of the article, and read and approved the final manuscript.

## Conflict of Interest

The authors declare that the research was conducted in the absence of any commercial or financial relationships that could be construed as a potential conflict of interest.

## Publisher's Note

All claims expressed in this article are solely those of the authors and do not necessarily represent those of their affiliated organizations, or those of the publisher, the editors and the reviewers. Any product that may be evaluated in this article, or claim that may be made by its manufacturer, is not guaranteed or endorsed by the publisher.

## References

[1] Pollock A Campbell P Cheyne J Cowie J Davis B McCallum J . Interventions to support the resilience and mental health of frontline health and social care professionals during and after a disease outbreak, epidemic or pandemic: a mixed methods systematic review. Cochrane Database Syst Rev. (2020) 11:CD013779. 10.1002/14651858.CD013779 33150970 PMC8226433

[2] World Health Organization Director-General's Opening Remarks at the Media Briefing on COVID-19-11 March 2020 [press release]. Geneva: World Health Organisation (2020).

[3] Matsuishi K Kawazoe A Imai H Ito A Mouri K Kitamura N . Psychological impact of the pandemic (H1N1) 2009 on general hospital workers in Kobe. Psychiatry Clin Neurosci. (2012) 66:353–60. 10.1111/j.1440-1819.2012.02336.x 22624741

[4] Lee SM Kang WS Cho AR Kim T Park JK. Psychological impact of the 2015 MERS outbreak on hospital workers and quarantined hemodialysis patients. Compr Psychiatry. (2018) 87:123–7. 10.1016/j.comppsych.2018.10.003 30343247 PMC7094631

[5] Lehmann M Bruenahl CA Löwe B Addo MM Schmiedel S Lohse AW . Ebola and psychological stress of health care professionals. Emerg Infect Dis. (2015) 21:913–4. 10.3201/eid2105.141988 25897490 PMC4412243

[6] Sahebi A Nejati-Zarnaqi B Moayedi S Yousefi K Torres M Golitaleb M. The prevalence of anxiety and depression among healthcare workers during the COVID-19 pandemic: an umbrella review of meta-analyses. Prog Neuropsychopharmacol Biol Psychiatry. (2021) 107:110247. 10.1016/j.pnpbp.2021.110247 33476692 PMC7817526

[7] Batra K Singh TP Sharma M Batra R Schvaneveldt N. Investigating the psychological impact of COVID-19 among healthcare workers: a meta-analysis. Int J Environ Res Public Health. (2020) 17:1–33. 10.3390/ijerph17239096 33291511 PMC7730003

[8] Kisely S Warren N McMahon L Dalais C Henry I Siskind D. Occurrence, prevention, and management of the psychological effects of emerging virus outbreaks on healthcare workers: rapid review and meta-analysis. BMJ. (2020) 369:m1642. 10.1136/bmj.m1642 32371466 PMC7199468

[9] Luo M Guo L Yu M Jiang W Wang H. The psychological and mental impact of coronavirus disease 2019 (COVID-19) on medical staff and general public - a systematic review and meta-analysis. Psychiatry Res. (2020) 291:113190. 10.1016/j.psychres.2020.113190 32563745 PMC7276119

[10] Pappa S Ntella V Giannakas T Giannakoulis VG Papoutsi E Katsaounou P. Prevalence of depression, anxiety, and insomnia among healthcare workers during the COVID-19 pandemic: a systematic review and meta-analysis. Brain Behav Immun. (2020) 88:901–7. 10.1016/j.bbi.2020.05.026 32437915 PMC7206431

[11] Serrano-Ripoll MJ Meneses-Echavez JF Ricci-Cabello I Fraile-Navarro D Fiol-deRoque MA Pastor-Moreno G . Impact of viral epidemic outbreaks on mental health of healthcare workers: a rapid systematic review and meta-analysis. J Affect Disord. (2020) 277:347–57. 10.1016/j.jad.2020.08.034 32861835 PMC7443314

[12] Huang Y Zhao N. Generalized anxiety disorder, depressive symptoms and sleep quality during COVID-19 outbreak in China: a web-based cross-sectional survey. Psychiatry Res. (2020) 288:112954. 10.1016/j.psychres.2020.112954 32325383 PMC7152913

[13] Zhang WR Wang K Yin L Zhao WF Xue Q Peng M . Mental health and psychosocial problems of medical health workers during the COVID-19 epidemic in China. Psychother Psychosom. (2020) 89:242–50. 10.1159/000507639 32272480 PMC7206349

[14] Greenberg N Docherty M Gnanapragasam S Wessely S. Managing mental health challenges faced by healthcare workers during covid-19 pandemic. BMJ. (2020) 368:m1211. 10.1136/bmj.m1211 32217624

[15] Arnetz JE Goetz CM Arnetz BB Arble E. Nurse reports of stressful situations during the COVID-19 pandemic: qualitative analysis of survey responses. Int J Environ Res Public Health. (2020) 17:1–12. 10.3390/ijerph17218126 33153198 PMC7663126

[16] Gupta S Sahoo S. Pandemic and mental health of the front-line healthcare workers: a review and implications in the Indian context amidst COVID-19. Gen Psychiatr. (2020) 33:e100284. 10.1136/gpsych-2020-100284 34192235 PMC7415074

[17] Robertson E Hershenfield K Grace SL Stewart DE. The psychosocial effects of being quarantined following exposure to SARS: a qualitative study of Toronto health care workers. Can J Psychiatry. (2004) 49:403–7. 10.1177/070674370404900612 15283537

[18] Park JS Lee EH Park NR Choi YH. Mental health of nurses working at a government-designated hospital during a MERS-CoV outbreak: a cross-sectional study. Arch Psychiatr Nurs. (2018) 32:2–6. 10.1016/j.apnu.2017.09.006 29413067 PMC7127092

[19] Galehdar N Kamran A Toulabi T Heydari H. Exploring nurses' experiences of psychological distress during care of patients with COVID-19: a qualitative study. BMC Psychiatry. (2020) 20:489. 10.1186/s12888-020-02898-1 33023535 PMC7538040

[20] McMahon SA Ho LS Brown H Miller L Ansumana R Kennedy CE. Healthcare providers on the frontlines: a qualitative investigation of the social and emotional impact of delivering health services during Sierra Leone's Ebola epidemic. Health Policy Plan. (2016) 31:1232–9. 10.1093/heapol/czw055 27277598 PMC5035780

[21] Waldman A Kaplan J. The Hospital System Sent Patients With Coronavirus Home to Die. Louisiana Legislators Are Demanding an Investigation. New Orleans: Propublica. (2020).

[22] Reynolds DL Garay JR Deamond SL Moran MK Gold W Styra R. Understanding, compliance and psychological impact of the SARS quarantine experience. Epidemiol Infect. (2008) 136:997–1007. 10.1017/S0950268807009156 17662167 PMC2870884

[23] National Institute for Clinical E. Post-Traumatic Stress Disorder: NICE Guideline. (2018). Available online at: www.nice.org.uk/guidance/ng116 (accessed February 16, 2021).

[24] Australian Government. National Mental Health Wellbeing Pandemic Response Plan. (2020). Available online at: https://www.mentalhealthcommission.gov.au/mental-health-and-wellbeing-pandemic-response-plan (accessed May 5, 2020).

[25] Whittemore R Knafl K. The integrative review: updated methodology. J Adv Nurs. (2005) 52:546–53. 10.1111/j.1365-2648.2005.03621.x 16268861

[26] Conn VS Rantz MJ. Research methods: managing primary study quality in meta-analyses. Res Nurs Health. (2003) 26:322–33. 10.1002/nur.10092 12884420

[27] National Institutes of Health. Quality Assessment Tool for Observational Cohort and Cross-Sectional Studies. (2020). Available online at: http://www.nhlbi.nih.gov/health-pro/guidelines/in-develop/cardiovascular-risk-reduction/tools/cohort (accessed April 27, 2020).

[28] Critical Appraisal Skills Programme. CASP (Qualitative) Checklist 2018. (2018). Available online at: https://casp-uk.net/wp-content/uploads/2018/01/CASP-Qualitative-Checklist-2018.pdf (accessed May 2, 2020).

[29] McArthur A Klugarova J Yan H Florescu S. Innovations in the systematic review of text and opinion. Int J Evid Based Healthc. (2015) 13:188–95. 10.1097/XEB.0000000000000060 26207851

[30] Page MJ McKenzie JE Bossuyt PM Boutron I Hoffmann TC Mulrow CD . Updating guidance for reporting systematic reviews: development of the PRISMA 2020 statement. J Clin Epidemiol. (2021) 134:103–12. 10.1016/j.jclinepi.2021.02.003 33577987

[31] Cheng W Zhang F Liu Z Zhang H Lyu Y Xu H . A psychological health support scheme for medical teams in COVID-19 outbreak and its effectiveness. Gen Psychiatr. (2020) 33:e100288. 10.1136/gpsych-2020-100288 34192236 PMC7462042

[32] Poonian J Walsham N Kilner T Bradbury E Brooks K West E. Managing healthcare worker well-being in an Australian emergency department during the COVID-19 pandemic. Emerg Med Austral. (2020) 32:700–2. 10.1111/1742-6723.13547 32386263 PMC7272835

[33] Ripp J Peccoralo L Charney D. Attending to the emotional well-being of the health care workforce in a New York City health system during the COVID-19 pandemic. Acad Med. (2020) 95:1136–9. 10.1097/ACM.0000000000003414 32282344 PMC7176260

[34] Donnelly PD Davidson M Dunlop N McGale M Milligan E Worrall M . Well-being during coronavirus disease 2019: a PICU practical perspective. Pediatr Crit Care Med. (2020) 21:e584–6. 10.1097/PCC.0000000000002434 32412984 PMC7402608

[35] Krystal JH Alvarado J Ball SA Fortunati FG Hu M Ivy ME . Mobilizing an institutional supportive response for healthcare workers and other staff in the context of COVID-19: the yale experience. Gen Hosp Psychiatry. (2021) 68:12–8. 10.1016/j.genhosppsych.2020.11.005 33254081 PMC7680059

[36] Caravella RA Deutch AB Noulas P Ying P Liaw KR-L Greenblatt J . Development of a virtual consultation-liaison psychiatry service: a multifaceted transformation. Psychiatric Ann. (2020) 50:279–87. 10.3928/00485713-20200610-02

[37] Zhang J Wu W Zhao X Zhang W. Recommended psychological crisis intervention response to the 2019 novel coronavirus pneumonia outbreak in China: a model of West China Hospital. Prec Clin Med. (2020) 3:3–8. 10.1093/pcmedi/pbaa006 PMC7107095 35960676

[38] Schulte EE Bernstein CA Cabana MD. Addressing faculty emotional responses during the coronavirus 2019 pandemic. J Pediatr. (2020) 222:13–4. 10.1016/j.jpeds.2020.04.057 32387715 PMC7204729

[39] Shen X Zou X Zhong X Yan J Li L. Psychological stress of ICU nurses in the time of COVID-19. Critic Care. Springer (2020) 24:1–3. 10.1186/s13054-020-02926-2 32375848 PMC7202793

[40] Lee S-H Juang Y-Y Su Y-J Lee H-L Lin Y-H Chao C-C. Facing SARS: psychological impacts on SARS team nurses and psychiatric services in a Taiwan general hospital. General Hospital Psychiatry. (2005) 27:352–8. 10.1016/j.genhosppsych.2005.04.007 16168796 PMC7132375

[41] Bernstein CA Bhattacharyya S Adler S Alpert JE. Staff emotional support at montefiore medical center during the COVID-19 pandemic. Jt Comm J Qual Patient Saf. (2021) 47:185–9. 10.1016/j.jcjq.2020.11.009 33353851 PMC7673209

[42] Davies C Campbell S Hutton J Morgan A Cherry MG. A summary of the rapid services changes made in response to staff psychological needs and maintaining care to those with cancer in light of COVID-19. Psycho-Oncol. (2020) 29:1393–4. 10.1002/pon.5449 32573022 PMC7362050

[43] Spray AM Patel NA Sood A Wu SX Simon NM Podbury R . Development of wellness programs during the COVID-19 pandemic response. Psychiatric Ann. (2020) 50:289–94. 10.3928/00485713-20200613-0133979296

[44] Wei E Segall J Villanueva Y Dang LB Gasca VI Gonzalez MP . Coping with trauma, celebrating life: reinventing patient and staff support during the COVID-19 pandemic. Health Aff. (2020) 39:1597–600. 10.1377/hlthaff.2020.00929 32673086

[45] Mellins CA Mayer LES Glasofer DR Devlin MJ Albano AM Nash SS . Supporting the well-being of health care providers during the COVID-19 pandemic: the CopeColumbia response. Gen Hosp Psychiatry. (2020) 67:62–9. 10.1016/j.genhosppsych.2020.08.013 33059217 PMC7480793

[46] Joseph E. Focusing on the mental health of nurses during the COVID-19 pandemic. N M Nurse. (2020) 65:12. Retrieved from https://www.nursingald.com/articles/27083-focusing-on-the-mental-health-of-nurses-during-the-covid-19-pandemic

[47] Cohen JK. UNC, Google team up on mental health app for healthcare workers. Mod Healthc. (2020) 50:4.

[48] Dursun OB Turan B Pakyurek M Tekin A. Integrating telepsychiatric services into the conventional systems for psychiatric support to health care workers and their children during COVID-19 pandemics: results from a national experience. Telemed eHealth. (2021) 2021:7. 10.1089/tmj.2020.0237 32821025

[49] DePierro J Katz CL Marin D Feder A Bevilacqua L Sharma V . Mount Sinai's center for stress, resilience and personal growth as a model for responding to the impact of COVID-19 on health care workers. Psychiatry Res. (2020) 293:113426. 10.1016/j.psychres.2020.113426 32861094 PMC7443339

[50] Ey S Soller M Moffit M. Protecting the well-being of medical residents and faculty physicians during the COVID-19 pandemic: making the case for accessible, comprehensive wellness resources. Global Adv Health Med. (2020) 9:2164956120973981. 10.1177/2164956120973981 33329941 PMC7720314

[51] Blake H Yildirim M Wood B Knowles S Mancini H Coyne E . COVID-well: evaluation of the implementation of supported wellbeing centres for hospital employees during the COVID-19 pandemic. Int J Environ Res Public Health. (2020) 17:1–22. 10.3390/ijerph17249401 33333913 PMC7768437

[52] Maunder R Hunter J Vincent L Bennett J Peladeau N Leszcz M . The immediate psychological and occupational impact of the 2003 SARS outbreak in a teaching hospital. Canadian Med Assoc J. (2003) 168:1245–51. Available online at: https://www.ncbi.nlm.nih.gov/pmc/articles/PMC154178/12743065 PMC154178

[53] Saqib A Rampal T. Quality improvement report: setting up a staff well-being hub through continuous engagement. BMJ Open Quality. (2020) 9:e001008. 10.1136/bmjoq-2020-001008 32826279 PMC7445343

[54] Chen Q Liang M Li Y Guo J Fei D Wang L . Mental health care for medical staff in China during the COVID-19 outbreak. Lancet Psychiatry. (2020) 7:e15–6. 10.1016/S2215-0366(20)30078-X 32085839 PMC7129426

[55] Gonzalez A Cervoni C Lochner M Marangio J Stanley C Marriott S. Supporting health care workers during the COVID-19 pandemic: Mental health support initiatives and lessons learned from an academic medical center. Psychol Trauma. (2020) 12(Suppl. 1):S168–70. 10.1037/tra0000893 32584111

[56] Gutkin PM Minneci MO Valenton J Kovalchuk N Chang DT Horst KC. Importance of a culture committee for boosting morale and maintaining a healthy work environment in radiation oncology. Adv Radiat Oncol. (2020) 5:1115–7. 10.1016/j.adro.2020.07.002 32838068 PMC7368646

[57] Nelson B Kaminsky DB. COVID-19's crushing mental health toll on health care workers. Cancer Cytopath. (2020) 128:597–8. 10.1002/cncy.22347 32885911

[58] Davis C Chong NK Oh JY Baeg A Rajasegaran K Elaine Chew CS. Caring for children and adolescents with eating disorders in the current COVID-19 pandemic: a Singapore perspective. J Adolesc Health. (2020) 67:131–4. 10.1016/j.jadohealth.2020.03.037 32381385 PMC7136888

[59] Liu S Yang L Zhang C Xiang YT Liu Z Hu S . Online mental health services in China during the COVID-19 outbreak. Lancet Psychiatry. (2020) 7:e17–8. 10.1016/S2215-0366(20)30077-8 32085841 PMC7129099

[60] Hall DL Millstein RA Luberto CM Perez GK Park ER. Responding to COVID-19 stress: disseminating mind-body resiliency approaches. Global Adv Health Med. (2020) 9:1–4. 10.1177/2164956120976554 33312763 PMC7716064

[61] Rosen B Preisman M Hunter J Maunder R. Applying psychotherapeutic principles to bolster resilience among health care workers during the COVID-19 pandemic. Am J Psychother. (2020) 73:144–8. 10.1176/appi.psychotherapy.20200020 32985915

[62] Aiello A Khayeri MY Raja S Peladeau N Romano D Leszcz M . Resilience training for hospital workers in anticipation of an influenza pandemic. J Contin Educ Health Prof. (2011) 31:15–20. 10.1002/chp.20096 21425355

[63] Amiel GE Ulitzur N. Caring for the caregivers: mental and spiritual support for healthcare teams during the COVID-19 pandemic and beyond. J Cancer Educ. (2020) 35:839–40. 10.1007/s13187-020-01859-2 32929697 PMC7490205

[64] Maunder RG Lancee WJ Mae R Vincent L Peladeau N Beduz MA . Computer-assisted resilience training to prepare healthcare workers for pandemic influenza: a randomized trial of the optimal dose of training. BMC Health Serv Res. (2010) 10:72. 10.1186/1472-6963-10-72 20307302 PMC2851711

[65] DeCaporale-Ryan L Goodman J Simning A Press-Ellingham L Williams L Hasselberg M. Addressing Skilled Nursing Facilities' COVID-19 Psychosocial Needs Via Staff Training and a Process Group Intervention. New York, NY: Elsevier B.V (2020). p. 894–5. 10.1016/j.jagp.2020.04.023 PMC7189870 32417025

[66] Rodriguez RM Medak AJ Baumann BM Lim S Chinnock B Frazier R . Academic emergency medicine physicians' anxiety levels, stressors and potential stress mitigation measures during the acceleration phase of the COVID-19 pandemic. Acad Emerg Med. (2020) 22:22. 10.1111/acem.14065 32569419 PMC7361565

[67] Azizoddin DR Gray KV Dundin A Szyld D. Bolstering clinician resilience through an interprofessional, web-based nightly debriefing program for emergency departments during the COVID-19 pandemic. J Interprofessional Care. (2020) 34:711–5. 10.1080/13561820.2020.1813697 32990108

[68] Wu K Wei X. Analysis of psychological and sleep status and exercise rehabilitation of front-line clinical staff in the fight against COVID-19 in China. Med Sci Monit Basic Res. (2020) 26:e924085. 10.12659/MSMBR.924085 32389999 PMC7241216

[69] Viswanathan R Myers MF Fanous AH. Support groups and individual mental health care via video conferencing for frontline clinicians during the COVID-19 pandemic. Psychosomatics. (2020) 61:538–43. 10.1016/j.psym.2020.06.014 32660876 PMC7308785

[70] Owens KM. Moving forward to nurture workforce resilience in crisis. Front Health Serv Manage. (2020) 37:14–9. 10.1097/HAP.0000000000000094 32842084

[71] Khee KS Lee LB Chai OT Loong CK Ming CW Kheng TH. The psychological impact of SARS on health care providers. Crit Care Shock. (2004) 7:99–106. Available online at: https://www.semanticscholar.org/paper/The-Psychological-impact-of-SARS-on-health-care-Khee-Lee/31dd73a903df3be7aca5b0bd68aafb5193a6989c34123910

[72] Monette DL Macias-Konstantopoulos WL Brown DFM Raja AS Takayesu JK. A video-based debriefing program to support emergency medicine clinician well-being during the COVID-19 pandemic. West J Emerg Med. (2020) 21:88–92. 10.5811/westjem.2020.8.48579 33052815 PMC7673898

[73] Siracusano A Di Lorenzo G Longo L Alcini S. Niolu CJCiE-EMDICPSGSESCI, a new edition of Web of Science. Building a resilient hospital in Tor Vergata: the role of emotional defusing for health care workers during COVID-19 pandemic. J Psychopath. (2020) 131–3. 10.36148/2284-0249-403

[74] Chan AO Huak CY. Psychological impact of the 2003 severe acute respiratory syndrome outbreak on health care workers in a medium size regional general hospital in Singapore. Occup Med. (2004) 54:190–6. 10.1093/occmed/kqh027 15133143 PMC7107861

[75] Datta SS Mukherjee A Ghose S Bhattacharya S Gyawali B. Addressing the mental health challenges of cancer care workers in LMICs during the time of the COVID-19 pandemic. JCO Glob Oncol. (2020) 2020:1490–3. 10.1200/GO.20.00470 33017180 PMC7605381

[76] He Z Chen J Pan K Yue Y Cheung T Yuan Y . The development of the 'COVID-19 psychological resilience model' and its efficacy during the COVID-19 pandemic in China. Int J Biol Sci. (2020) 16:2828–34. 10.7150/ijbs.50127 33061799 PMC7545720

[77] Geoffroy PA Le Goanvic V Sabbagh O Richoux C Weinstein A Dufayet G . Psychological support system for hospital workers during the Covid-19 outbreak: rapid design and implementation of the Covid-Psy hotline. Front Psychiatry. (2020) 11:511. 10.3389/fpsyt.2020.00511 32670100 PMC7326137

[78] Matthewson J Tiplady A Gerakios F Foley A Murphy E. Implementation and analysis of a telephone support service during COVID-19. Occup Med. (2020) 70:375–81. 10.1093/occmed/kqaa095 32449754 PMC7313817

[79] Maldonato NM Bottone M Chiodi A Continisio GI De Falco A Duval M . A mental health first aid service in an italian university public hospital during the coronavirus disease 2019 outbreak. Sustainability. (2020) 12:12. 10.3390/su12104244

[80] Kameno Y Hanada A Asai D Naito Y Kuwabara H Enomoto N . Individual psychotherapy using psychological first aid for frontline nurses at high risk of psychological distress during the COVID-19 pandemic. Psychiatry Clin Neurosci. (2021) 75:25–7. 10.1111/pcn.13170 33131157

[81] Chung JPY Wai-song Y. Staff mental health self-assessment during the COVID-19 outbreak. East Asian Arch Psychiatry. (2020) 30:34. 10.12809/eaap2014 32229646

[82] Machado AS Pereira E Grangeia R Norton P. Mental health support to health care workers during COVID-19 pandemic: is the front line necessarily the priority line? J Occup Environ Med. (2020) 62:e677–8. 10.1097/JOM.0000000000002011 32858557

[83] Buselli R Baldanzi S Corsi M Chiumiento M Del Lupo E Carmassi C . Psychological care of health workers during the COVID-19 outbreak in italy: preliminary report of an occupational health department (AOUP) responsible for monitoring hospital staff condition. Sustainability. (2020) 12:5039. 10.3390/su12125039

[84] Jo SH Koo BH Seo WS Yun SH Kim HG. The psychological impact of the coronavirus disease pandemic on hospital workers in Daegu, South Korea. Compr Psychiatry. (2020) 103:152213. 10.1016/j.comppsych.2020.152213 33096399 PMC7553934

[85] Giordano F Scarlata E Baroni M Gentile E Puntillo F Brienza N . Receptive music therapy to reduce stress and improve wellbeing in Italian clinical staff involved in COVID-19 pandemic: a preliminary study. Arts Psychother. (2020) 70:101688. 10.1016/j.aip.2020.101688 32834302 PMC7361107

[86] Torricelli L Poletti M Raballo A. Managing COVID-19-related psychological distress in health workers: field experience in northern Italy. Psychiatry Clin Neurosci. (2021) 75:23–4. 10.1111/pcn.13165 33084145

[87] Bowman C Branjerdporn G Turner K Kamara M Tyagi N Delos Reyes NT . The impact of viral epidemics and pandemics on acute mental health service use: an integrative review. Health Psychol Rev. (2021) 15:1–33. 10.1080/17437199.2021.1886864 33550940

