# Supplementary material for: Interventional Response of Hospital and Health Services to the Mental Health Effects of Viral Outbreaks on Health Professionals
Source: Front Psychiatry. 2022 Feb 22;13:812365. doi: 10.3389/fpsyt.2022.812365 (PMC8902291; doi:10.3389/fpsyt.2022.812365)
Supplement: Supplementary file 1 [file Table_1.DOCX]

**Supplementary Table 1**

*Key aspects of included papers*

| Article, Pandemic, Country | Article type | Intervention | Outcomes |
| --- | --- | --- | --- |
| 1. **Aiello et al., 2011.**   H1N1, USA | Qualitative | One-hour resilience training session in anticipation of influenza pandemic. | N=1250. More participants felt prepared to deal with a pandemic after the session. |
| 1. **Amiel & Ulitzur, 2020.**   COVID-19, Israel | Commentary | Resilience program to understand compassion, fatigue, and the impact of stress. Conducted three sessions per week. | Positive impacts from program lasted 3 months post-workshop. Further workshops desired. |
| 1. **Azizoddin et al., 2020.**   COVID-19, USA | Qualitative | Six-week interpersonal debriefing program. | Discussed challenges of social distancing, scarce resources, sense of helplessness, & burnout. |
| 1. **Bernstein et al., 2021.**   COVID-19, USA. | Commentary | Psychoeducational resources (e.g., webinars). Peer support outreach. Psychological first aid. Hotline. Food. Telehealth psychotherapy with pharmacotherapy treatment. Wellness programs (e.g., meditation and art). Clergy support. | Psychoeducation resources, peer support, and psychological first aid and food were well-received. |
| 1. **Blake et al., 2020.** COVID-19, United Kingdom. | Cross-sectional | Wellbeing centres with relaxation rooms in hospital, staffed by volunteers trained in psychological first aid. | 14,934 facility visits. Of 819 staff, wellbeing was higher in those that accessed a centre. |
| 1. **Buselli et al., 2020.**   COVID-19, Italy | Cross-sectional | Dedicated email account for psychological first aid, cognitive behavioural therapy and mindfulness-based and relaxation techniques. Psychoeducational leaflets. | Positive feedback received by staff (e.g., share common experiences and concerns, mutual support). |
| 1. **Caravella et al., 2020.**   COVID-19, USA. | Commentary | Virtual consultation and liaison psychiatric services delivered by psychiatric nurses. | Positive feedback received by staff. |
| 1. **Chan & Huak, 2004.**   SARS, Singapore. | Cross-sectional | Crisis counselling program led by voluntary peers. Addressed staff feedback. | Positive feedback received by staff. |
| 1. **Chen et al., 2020.**  COVID-19, China. | Commentary | Online psychoeducation courses. Hotline. Psychological group interventions. Leisure activities. Counsellors. Accommodation. Daily living supplies. Videos to family. Pre-job training. Security staff. Protective equipment guidelines. | Initial reluctance to engage in psychological interventions. Practical interventions appreciated. |
| 1. **Cheng et al., 2020.** COVID-19, China. | Pilot | Daily measurement of mood. Daily mood broadcasts to promote positivity. Daily online peer-group activity. Balint groups. After-work support team. | N=155. Improved mood reported. High attention and effectiveness ratings. |
| 1. **Chung & Wai-Song, 2020.** COVID-19, Hong Kong | Commentary | Mental health survey with option to request psychological support and receive psychotherapy by psychiatric nurse. | N=69. 3% requested psychological support by nurse. |
| 1. **Cohen et al., 2020**.   COVID-19, USA. | Commentary | Mobile application provided mental health resources, administered psychological assessments, and notified hospital management of distress. | Positive feedback received by staff. |
| 1. **Datta et al., 2020**.  COVID-19, India | Commentary | Staff meetings to address anxieties related to COVID-19, led by psychiatrists, a microbiologist, and a public health specialist. Psychological first aid. | Positive feedback received by staff. |
| 1. **Davies et al., 2020.**  COVID-19, United Kingdom. | Commentary | Wellbeing hubs. Psychological support. Weekly bulletin providing psychological wellbeing resources. Telephone and video calls. | Positive feedback received by staff. Low uptake on weekly bulletin. |
| 1. **DeCaporale-Ryan et al., 2020**. COVID-19, USA | Commentary | Virtual sessions discussing interventions to reduce isolation and acknowledge challenges faced. | N=67. Sessions helpful for self-care, affirming experience, and patient care. |
| 1. **DePierro et al., 2020**.   COVID-19, USA. | Commentary | Mental health mobile application. Peer-led workshops regarding resilience. Remote mental health screening. Mental health service provision. | Positive feedback received by staff. |
| 1. **Donnelly et al., 2020.**   COVID-19, United Kingdom. | Commentary | Tea breaks. Celebrating small achievements. Team badges. Online calls. ‘Take a minute’ room. Peer support network. Debriefing with mental health team. | Positive feedback received by staff. |
| 1. **Dursun et al., 2021.**  COVID-19, Turkey. | Cross-sectional | Mobile application to request mental health support. Telehealth and face-to-face consultations with mental health professionals. | N=879. High satisfaction. 86.6% reported needs met. 1.4% required face-to-face support. |
| 1. **Ey et al., 2020.**   COVID-19, USA. | Commentary | Individual counselling. Psychotropic medication. Telehealth visits. | Positive feedback received by staff. |
| 1. **Geoffroy et al., 2020.**  COVID-19, France | Ecological | Telephone hotline. Individual psychology sessions. Referral to other psychological supports such as cognitive behavioural therapy and psychiatry. | Reasons for calls: anxiety, exhaustion, trauma, psychosis, insomnia, anger & depression. |
| 1. **Giordano et al., 2020.**  COVID-19, Italy | Pilot | Receptive music therapy by trained music therapists with three 15-20 min playlists of breathing, energy and serenity. | Reduced intensity of tiredness, sadness, fears and worry. |
| 1. **Gonzalez et al., 2020.**  COVID-19, USA | Commentary | 24/7 employee respite area for resting, showering, receiving emotional support, eating, and relaxing with aromatherapy, music, television, and adult colouring books. | Positive feedback received by staff. |
| 1. **Gutkin et al., 2020.**   COVID-19, USA | Cross-sectional | Virtual social gatherings and interactions. Care packages. Grocery store gift cards. Boxed lunches. Weekly podcasts. | Increased morale. Gift cards, meals, and care packages rated as “extremely helpful” by 59%. |
| 1. **Hall et al., 2020.**   COVID-19, USA | Commentary | Resiliency program of eight 90-minute sessions to improve stress awareness, coping skills, and relaxation (e.g., mindfulness, self-empathy, humour). | Positive feedback received by staff. |
| 1. **He et al., 2020.**  COVID-19, China. | Cross-sectional | Hotline consultation. Online video psychotherapy. Onsite crisis intervention | Improved problem solving and relieved stress. |
| 1. **Jo et al., 2020.**   COVID-19, South Korea | Cross-sectional | Telepsychiatry consultations. Administered assessments [Mini International Neuropsychiatric Interview (MINI) and the Clinical Global Impressions-Severity (CGI-S)] | N=54. Psychological symptoms improved after 2 weeks. |
| 1. **Joseph et al., 2020.**   COVID-19, USA. | Commentary | Virtual peer-to-peer support to share experiences and receive support.  Mental health resources. | Positive feedback received by staff. |
| 1. **Kameno et al., 2020**.   COVID-19, Japan | Cross-sectional | High-risk individuals were provided individual psychotherapy by mental health nurse. | Psychological distress improved  compared to those without intervention. |
| 1. **Khee et al., 2004**.   SARS, Singapore | Qualitative | In-person supportive group therapy sessions developed by psychologists. | Discussed issues such as grief, anger, death, spirituality, failure and death. |
| 1. **Krystal et al., 2021.**  COVID-19, USA. | Commentary | Buddy system. Virtual support huddles. Daily rounding. Inter-departmental leadership meetings. 24/7 hotlines. Employee assistance programs. Wellness checks. Meals for caregivers. Housing. Rest areas. | Positive feedback received by staff. |
| 1. **Lee et al., 2005.**  SARS, Taiwan. | Cross-sectional | Individual psychotherapy. Debriefing groups to discuss stressors and coping strategies. Counselling hotline. Psychoeducation. | Psychiatric services and debriefing groups were highly effective. Improvements suggested. |
| 1. **Liu et al., 2020.**  COVID-19, China. | Commentary | Digital mental health support. Online psychological support services via WeChat monitored suicide risk and notified support services as required. | Positive feedback received by staff.  50.7% staff described as having depression. |
| 1. **Machado et al., 2020.**   COVID-19, Portugal | Commentary | Three-level mental health intervention with support via mobile electronic application, psychiatry appointment, and telephone call. | Triggers, anxiety, insomnia, compulsive eating, amotivation, & poor concentration were identified. |
| 1. **Maldonato et al., 2020.**   COVID-19, Italy | Commentary | Psychological first aid telephone service. | Anxiety and fear of contagion motivated staff to seek psychological help. |
| 1. **Mathewson et al., 2020.**   COVID-19, United Kingdom | Ecological | Telephone service by the counselling and psychology team to provide information, and refer to half-hour support call with mental health practitioner, as required. | Discussed anxiety support (29%), clarification of guidance (68%), and non-COVID related (3%). |
| 1. **Maunder et al., 2003**.  SARS, Canada. | Qualitative | Anxiety/stress psychoeducation material. Psychiatry support offered at request of nurse manager. Drop-in support centre for relaxation. Telephone support line. | Positive feedback received by staff. |
| 1. **Maunder et al., 2010.**  SARS, Canada. | Pilot study | Computer-assisted resilience training of short (7 sessions, 111 mins), medium (12 sessions, 158 minutes), or long (17 sessions, 223 min) dosages. | Confidence in support and training, pandemic self-efficacy and interpersonal problems improved. |
| 1. **Mellins et al., 2020**   COVID-19, Columbia | Qualitative | Peer support program. Stress and coping strategies. Therapies (e.g., problem-solving therapy, cognitive behavioural therapy, and acceptance and commitment therapy). | Emotional distress decreased. Program was recommended. |
| 1. **Monette et al., 2020.**   COVID-19, USA | Pilot study | Debrief sessions conducted weekly, for one hour, via videoconferencing, with expert facilitator. | Positive feedback about debriefing with members of the same role group, and using Zoom. |
| 1. **Nelson & Kaminsky, 2020.** COVID-19, USA. | Commentary | Resilience and stress management programs. Eye movement desensitization and reprocessing therapy. | Positive feedback received by staff. |
| 1. **Owens, 2020.**   COVID-19, USA | Commentary | Virtual support including resources and tools. Video-conferencing sessions to discuss concerns and hopes. | Topics discussed included anxieties of life-and-death decisions, and daily ethical considerations. |
| 1. **Poonian et al., 2020**.  COVID-19, Australia. | Commentary | Daily communication by leadership team. Staff rest area with mindfulness and yoga sessions. Appropriate rostering. Vacation periods. Psychological first aid. Wellbeing drop-in sessions. Skills-based drills. | Positive feedback received by staff. |
| 1. **Ripp et al., 2020.**  COVID-19, USA | Commentary | Daily living needs supplies. Reliable and reassuring messages. Robust psychosocial and mental health support options. | Positive feedback received by staff. |
| 1. **Rodriguez et al., 2020.**   COVID-19, Spain | Cross-sectional | Twice daily on-site mindfulness program. | N = 150. Stress reduction of 8.4/10. |
| 1. **Rosen et al., 2020.**   COVID-19, Canada | Commentary | Psychiatrist led staff huddles twice a week to improve resilience and coping skills. | Positive feedback received by staff. |
| 1. **Saqib & Rampal, 2020.**   COVID-19, United Kingdom. | Commentary | Staff wellbeing hub outside hospital providing quiet space for de-stressing. Mindfulness sessions. Yoga sessions. | 33-46 mins were spent in hub. Improved mood reported. |
| 1. **Schulte et al., 2020.**   COVID-19, USA. | Commentary | Group video calls lasting one hour to discuss coping, lesson learned and challenges. | Positive feedback. Discussed fears, ethical dilemmas, and coping. |
| 1. **Shen et al., 2020.**  COVID-19, China. | Commentary | Psychological assessments and interventions. Relaxation exercises (e.g., drawing, singing, exercising). Peer support. Virtual team morale discussions. Mental health training. Individual/group psychotherapy via online & phone. Social support. | Positive feedback received by staff. |
| 1. **Siracusano et al., 2020.**  COVID-19, Italy | Cross-sectional | In-person emotional defusing through group sessions with those experiencing significant trauma. | Positive feedback received by staff. |
| 1. **Spray et al., 2020.**   COVID-19, USA | Commentary | Virtual drop-in groups. Posters with coping cards, stressors & helpline numbers. Support groups consisting of psychiatrists, psychologist, & senior residents. | Positive feedback received by staff. |
| 1. **Torricelli et al., 2020.**   COVID-19, Italy | Commentary | Eye-movement desensitisation and reprocessing technique led by trained psychotherapists. | Positive feedback received by staff. 1.5-hour sessions with 12-15 participants. |
| 1. **Viswanathan et al., 2020**. COVID-19, USA | Qualitative | Individual and group counselling sessions. Behavioural health hotline. Intranet page with resources. Webinars on resilience, wellness topics, grief, and mourning. | Positive feedback received by staff. |
| 1. **Wei et al., 2020**   COVID-19, USA | Commentary | Individual and group counselling sessions. Hotline. Intranet page with resources. Psychoeducational webinars. Respite rooms. Coordinated wellness rounds. Temporary accommodation. Complimentary day care. Free meals. Groceries for families. Transportation vouchers. Replacement scrubs. | Positive feedback received by staff. |
| 1. **Wu & Wei, 2020**   COVID-19, China | Case-control | At-home exercise rehabilitation program including yoga, tai chi, and qigong. | Reduced somatization, depression, post-traumatic stress symptoms and sleep disturbances. |
| 1. **Zhang et al., 2020.**   COVID-19, China. | Commentary | Online social support. Hotlines and online chat with mental health clinicians. Psychological rescue team for crisis intervention. Mental health training. | Positive feedback received by staff. |

USA = United States of America.
