# Supplementary material for: Interventional Response of Hospital and Health Services to the Mental Health Effects of Viral Outbreaks on Health Professionals
Source: Front Psychiatry. 2022 Feb 22;13:812365. doi: 10.3389/fpsyt.2022.812365 (PMC8902291; doi:10.3389/fpsyt.2022.812365)
Supplement: Supplementary file 2 [file Table_2.DOCX]

**Supplementary Table 2**

*Quality assessment results based on the National Heath Lung, and Blood Institute tool for cohort and cross-sectional studies*

| Study | 1. Was the research question or objective in this paper clearly stated? | 2. Was the study population clearly specified and defined? | 3. Was the participation rate of eligible persons at least 50%? | 4. Were all the subjects selected or recruited from the same or similar populations (including the same time period)? Were inclusion/exclusion criteria for being in the study prespecified and applied uniformly to all participants? | 5. Was a sample size justification, power description, or variance and effect estimates provided? | 6. For the analyses in this paper, were the exposure(s) of interest measured prior to the outcome(s) being measured? | 7. Was the timeframe sufficient so that one could reasonably expect to see association between exposure and outcome if it existed? | 8. Examination of different levels of the exposure related to the outcome? | 9. Exposure measures clearly defined, valid, reliable, and implemented consistently across all participants? | 10. Exposure assessed more than once | 11. Were the outcome measures (dependent variables) clearly defined, valid, reliable, and implemented consistently across all study participants? | 12. Outcome assessors blinded to exposure status? | 13.Was loss to follow-up after baseline <20%? | 14. Adjustment for Confounding variables and adjusted sig for their impact on relationship between exposure and outcome? |
| --- | --- | --- | --- | --- | --- | --- | --- | --- | --- | --- | --- | --- | --- | --- |
| 1. Blake et al., 2020 | Yes | Yes | No | Yes | No | No | Yes | No | Yes | No | Yes | No | N/A | No |
| 2. Buselli et al., 2020 | Yes | Yes | No | Yes | No | No | No | No | Yes | No | NR | No | No | No |
| 3. Chan & Huak, 2004 | Yes | Yes | N/A | Yes | N/A | No | NR | NR | N/R | NR | NR | No | No | No |
| 4. Cheng et al., 2020 | Yes | Yes | Yes | Yes | No | No | Yes | No | N/A | No | Yes | No | N/A | No |
| 5. Dursun et al., 2020 | Yes | Yes | No | Yes | Yes | No | No | No | Yes | No | Yes | No | No | No |
| 6. Geoffroy et al., 2020 | Yes | Yes | N/A | Yes | No | No | N/A | No | Yes | No | Yes | No | N/A | No |
| 7. Gutkin et al., 2020 | Yes | Yes | No | Yes | No | Yes | No | No | No | No | Yes | No | N/A | No |
| 8. He et al., 2020 | Yes | Yes | No | Yes | No | No | No | No | Yes | No | Yes | No | N/A | No |
| 9. Jo et al., 2020 | Yes | Yes | No | Yes | No | No | N/A | Yes | N/A | No | Yes | No | No | No |
| 10. Kameno et al., 2020 | Yes | Yes | No | Yes | No | No | Yes | No | No | No | Yes | No | No | No |
| 11. Lee et al. 2005 | Yes | Yes | Yes | NR | No | No | Yes | No | No | No | Yes | No | N/A | No |
| 12. Matthewson et al., 2020 | Yes | Yes | No | Yes | No | No | N/A | No | N/A | No | Yes | N/A | N/A | No |
| 13. Rodriguez et al., 2020 | Yes | No | No | Yes | No | Yes | Yes | No | Yes | No | Yes | No | No | Yes |
| 14. Siracusano et al, 2020 | Yes | Yes | No | Yes | No | No | Yes | No | No | No | Yes | No | No | No |

**Supplementary Table 3**

*Quality assessment results based on the National Heath Lung, and Blood Institute tool for pre-post studies (no control)*

| Study | 1. Was the study question or objective clearly stated? | 2. Were eligibility/selection criteria for the study population pre-specified and clearly described? | 3. Were the participants in the study representative of those who would be eligible for the test/service/  intervention in the general or clinical population of interest? | 4. Were all eligible participants that met the pre-specified entry criteria enrolled? | 5. Was the sample size sufficiently large to provide confidence in the findings? | 6. Was the test/service/intervention clearly described and delivered consistently across the study population? | | 7. Were the outcome measures prespecified, clearly defined, valid, reliable, and assessed consistently across all study participants? | 8. Were the people assessing the outcomes blinded to the participants' exposures/  interventions? | 9. Was the loss to follow-up after baseline 20% or less? Were those lost to follow-up accounted for in the analysis? | 10. Did the statistical methods examine changes in outcome measures from before to after intervention? Were statistical tests done that provided p values for the pre-to-post changes? | | 11. Were outcome measures of interest taken multiple times before the intervention and multiple times after the intervention (i.e., did they use an interrupted time-series design)? | 12. If the intervention was conducted at a group level (e.g., a whole hospital, a community, etc.) did the statistical analysis take into account the use of individual-level data to determine effects at the group level? |
| --- | --- | --- | --- | --- | --- | --- | --- | --- | --- | --- | --- | --- | --- | --- |
| 1. Giordano et al., 2020 | Yes | Yes | Yes | NR | No | Yes | Yes | | N/A | No | Yes | No | | N/A |
| 2. Maunder et al.2010 | Yes | Yes | Yes | Yes | NR | Yes | Yes | | N/A | No | Yes | No | | N/A |
| 3. Monette et al., 2020 | Yes | No | Yes | Yes | NR | Yes | Yes | | N/A | No | No | No | | N/A |

**Supplementary Table 4**

*Quality assessment results based on the National Heath Lung, and Blood Institute tool for case-control studies*

| Study | 1. Was the research question or objective in this paper clearly stated and appropriate? | 2. Was the study population clearly specified and defined? | 3. Did the authors include a sample size justification? | 4. Were controls selected or recruited from the same or similar population that gave rise to the cases (including the same timeframe)? | 5. Were the definitions, inclusion and exclusion criteria, algorithms or processes used to identify or select cases and controls valid, reliable, and implemented consistently across all study participants? | 6. Were the cases clearly defined and differentiated from controls? | 7. If less than 100 percent of eligible cases and/or controls were selected for the study, were the cases and/or controls randomly selected from those eligible? | 8. Was there use of concurrent controls? | 9. Were the investigators able to confirm that the exposure/risk occurred prior to the development of the condition or event that defined a participant as a case? | 10. Were the measures of exposure/risk clearly defined, valid, reliable, and implemented consistently (including the same time period) across all study participants? | 11. Were the assessors of exposure/risk blinded to the case or control status of participants? | 12. Were key potential confounding variables measured and adjusted statistically in the analyses? If matching was used, did the investigators account for matching during study analysis? |
| --- | --- | --- | --- | --- | --- | --- | --- | --- | --- | --- | --- | --- |
| 1. Wu 2020 | Yes | Yes | No | Yes | Yes | Yes | Yes | Yes | N/A | No | No | No |

**Supplementary Table 5**

*Quality assessment results based on the Critical Appraisal Skills Programme (CASP) qualitative checklist*

| Study | 1. Aims clearly stated | 2. Is a qualitative methodology appropriate? | 3. Was the research design appropriate to address the aims of the research? | 4. Was the recruitment strategy appropriate to aims of the research? | 5. Was the data collected in a way that addressed the research issue? | 6. Has the relationship between researcher and participants been considered? | 7. Have ethical issues been taken into consideration? | 8. Was the data analysis sufficiently rigorous? | 9. Is there a clear statement of findings? | 10. How valuable is the research? |
| --- | --- | --- | --- | --- | --- | --- | --- | --- | --- | --- |
| 1. Aiello et al., 2011 | Yes | Yes | Yes | No | Yes | No | Yes | No | Yes | Yes |
| 2. Azizoddin et al., 2020 | Yes | Yes | Yes | Yes | Yes | No | Yes | No | Yes | Yes |
| 3. Khee et al., 2004 | Yes | Yes | Yes | Yes | No | No | No | No | Yes | No |
| 4. Maunder et al., 2003 | Yes | Yes | Yes | Yes | Yes | No | No | No | Yes | Yes |
| 5. Mellins et al. (2020) | Yes | Yes | Yes | Yes | Yes | No | No | No | Yes | Yes |
| 6. Viswanathan et al., 2020 | Yes | Yes | Yes | Yes | Yes | No | No | No | Yes | Yes |

**Supplementary Table 6**

*Quality assessment results based on the Joanna Briggs Institute (JBI) checklist for text and opinion papers*

| Study | 1. Is the source of the opinion clearly identified? | 2. Does the source of opinion have standing in the field of expertise? | 3. Are the interests of the relevant population the central focus of the opinion? | 4. Is the state position the result of an analytical process, and is there logic in the opinion expressed? | 5. Is there reference to the extant literature? | 6. Is any incongruence with the literature/sources logically defended? |
| --- | --- | --- | --- | --- | --- | --- |
| 1. Amiel & Ulitzur, 2020 | Yes | Yes | Yes | Yes | Yes | No |
| 2. Bernstein et al., 2020 | Yes | Yes | Yes | Yes | Yes | No |
| 3. Caravella et al., 2020 | Yes | Yes | Yes | Yes | Yes | No |
| 4. Chen et al., 2020. | Yes | Yes | Yes | Yes | Yes | No |
| 5. Chung & Wai-Song, 2020. | Yes | Yes | Yes | Yes | Yes | No |
| 6. Cohen et al., 2020 | NR | NR | Yes | Yes | No | No |
| 7. Datta et al., 2020 | Yes | Yes | Yes | Yes | Yes | No |
| 8. Davies et al, 2020 | Yes | Yes | Yes | Yes | No | No |
| 9. DeCaporale-Ryan et al., 2020 | Yes | Yes | Yes | Yes | No | No |
| 10. DePierro et al., 2020 | Yes | Yes | Yes | Yes | No | No |
| 11. Donnelly et al., 2020 | Yes | Yes | Yes | Yes | Yes | No |
| 12. Ey et al., 2020 | Yes | Yes | Yes | Yes | Yes | No |
| 13. Gonzalez et al., 2020 | Yes | Yes | Yes | Yes | Yes | No |
| 14. Hall et al., 2020 | Yes | Yes | Yes | Yes | Yes | No |
| 15. Joseph et al., 2020 | Yes | Yes | Yes | Yes | Yes | No |
| 16. Krystal et al., 2021 | Yes | Yes | Yes | Yes | Yes | No |
| 17**.** Liu et al., 2020 | Yes | Yes | Yes | Yes | Yes | No |
| 18. Machado et al., 2020 | Yes | Yes | Yes | Yes | Yes | No |
| 19. Maldonato et al., 2020 | Yes | Yes | Yes | Yes | Yes | No |
| 20. Nelson & Kaminsky, 2020 | Yes | Yes | Yes | Yes | No | No |
| 21. Owens, 2020 | Yes | Yes | Yes | Yes | Yes | No |
| 22. Poonian et al., 2020 | Yes | Yes | Yes | Yes | Yes | No |
| 23. Ripp et al., 2020 | Yes | Yes | Yes | Yes | Yes | No |
| 24. Rosen et al., 2020 | Yes | Yes | Yes | Yes | Yes | No |
| 25. Saqib & Rampal, 2020 | Yes | Yes | Yes | Yes | Yes | No |
| 26. Schulte (2020) | Yes | Yes | Yes | Yes | Yes | No |
| 27. Shen (2020) | Yes | Yes | Yes | Yes | Yes | No |
| 28. Spray et al., 2020 | Yes | Yes | Yes | Yes | Yes | No |
| 29. Torricelli et al., 2021 | Yes | Yes | Yes | Yes | Yes | Yes |
| 30. Wei et al., 2020 | Yes | Yes | Yes | Yes | Yes | No |
| 31. Zhang et al. (2020) | Yes | Yes | Yes | Yes | Yes | No |
