# Supplementary material for: Interventional Response of Hospital and Health Services to the Mental Health Effects of Viral Outbreaks on Health Professionals
Source: Front Psychiatry. 2022 Feb 22;13:812365. doi: 10.3389/fpsyt.2022.812365 (PMC8902291; doi:10.3389/fpsyt.2022.812365)
Supplement: Supplementary file 3 [file Data_Sheet_1.docx]

**Figure 1**

*PRISMA Flowchart describing process of identification, screening, and inclusion*

Records removed
*before screening*:

Duplicate records removed

(n = 2450)

Records identified from:

Databases (n = 7944)

Hand searching (n = 6)

**Identification**

Records screened

(n = 5500)

Records excluded by human

(n = 4954)

Reports sought for retrieval

(n = 546)

Reports not retrieved

(n = 0)

**Screening**

Reports excluded:

Not in English (n = 7)

Wrong population (n = 20)

No intervention (n = 462)

Duplicates (n = 2)

Reports assessed for eligibility

(n = 546)

Studies included in review

(n = 55)

**Included**
